# Supplementary figures and images for: activin-2 is required for regeneration of polarity on the planarian anterior-posterior axis
Source: PLoS Genet. 2021 Mar 29;17(3):e1009466. doi: 10.1371/journal.pgen.1009466 (PMC8057570; doi:10.1371/journal.pgen.1009466)

Supplemental Figure 1

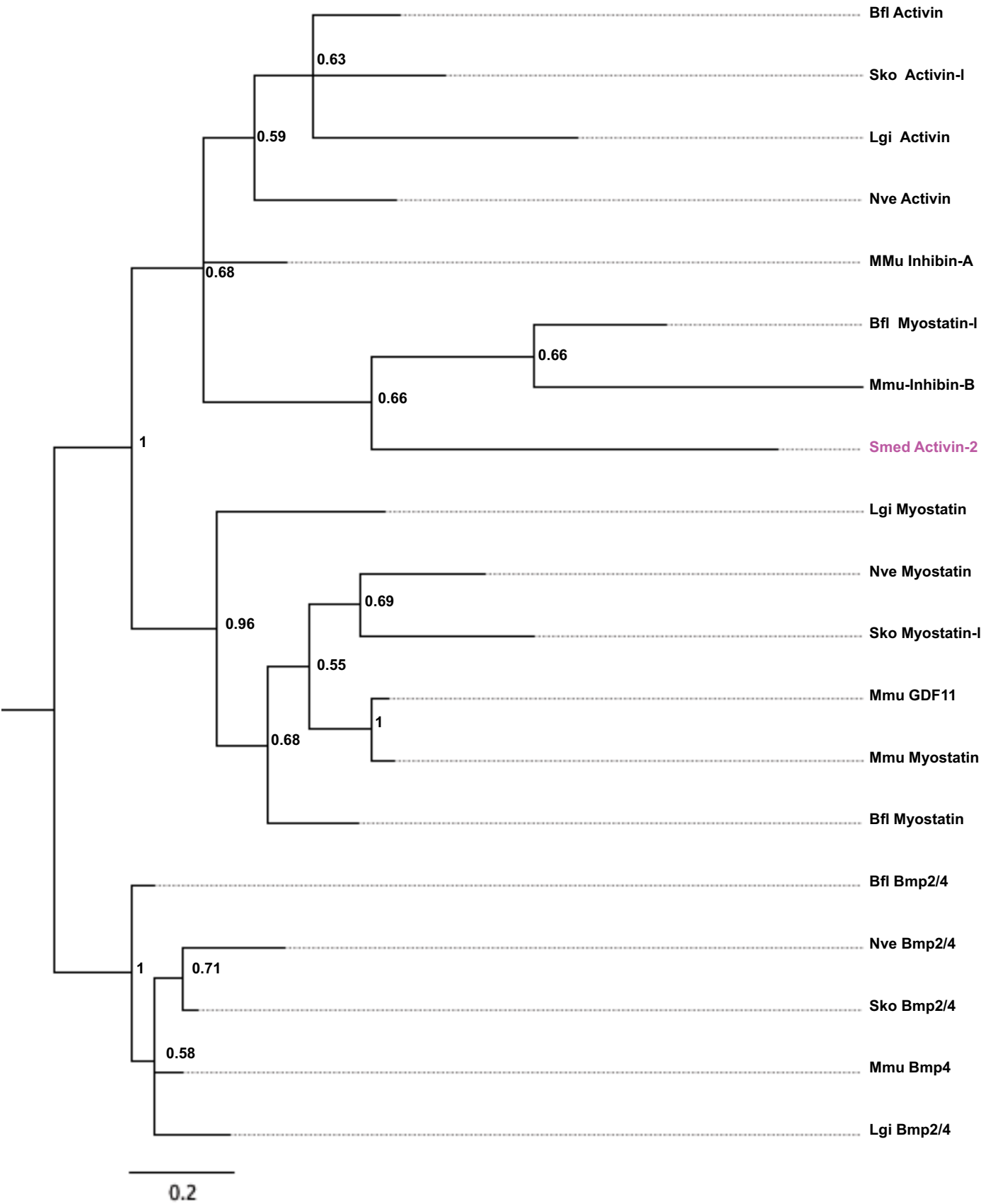

Supplement: S1 Fig — Related to Fig 1. (A) Phylogenetic tree for the placement of Schmidtea mediterranea activin-2. Bayesian analysis of TGF- superfamily ligand proteins with a focus on Activins and Myostatins, where BMP-2/4 is used as an outgroup across species. Percent posterior probability is indicated at nodes. Smed (Schmidtea mediterranea), Nve (Nematostella vectenesis), Mmu (Mus musculus), Bfl (Branchiostoma floridae), Sko (Saccoglossus kowalevskii), Lgi (Lottia gigantea). In Mus musculus genes that contribute to Activin proteins are called inhibin, we used this nomenclature in the tree. Protein sequences are provided in S1 Data. (PDF) [file pgen.1009466.s001.pdf]

## Supplemental Figure 2

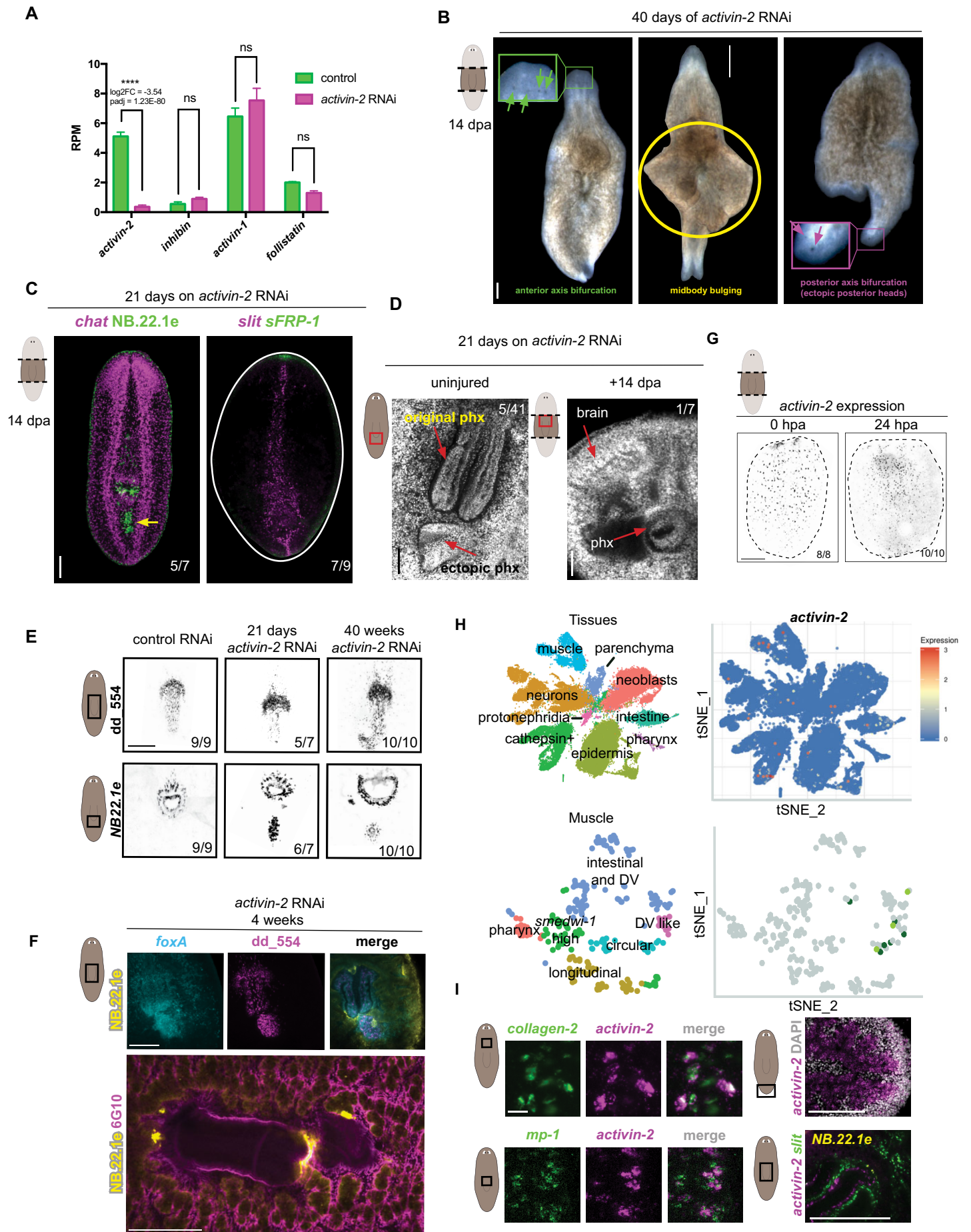

Supplement: S2 Fig — Related to Fig 1. (A) RNAi control. Transcript abundance for TGF-β ligands and follistatin from bulk sequencing expression data in control and activin-2 RNAi animals. RPM is reads per million. Data is three replicates of a post-pharyngeal fragment. Related to S2 Data. (B) Regenerating activin-2 RNAi animals after 40 days of RNAi, and 14 days post amputation. The three representative images show examples of (Left) anterior axis bifurcation, (Middle) parapharyngeal bulging indicative of ectopic pharyngeal tissue; (Right) posterior axis bifurcation and loss of polarity resulting in ectopic posterior heads. (C) Negative results related to 1C. FISH shows (left) CNS (chat+) and mouth and esophagus (NB.22.1e) and (right) PCG expression (midline slit+; anterior sFRP-1+). Yellow arrow shows ectopic posterior mouth tissue. (D) DAPI shows intact (Left) and regenerated (Right) animals develop ectopic pharynges. Red arrows show anatomy. Phx = pharynx. (E) Intact activin-2 RNAi animals develop ectopic pharynges (Top) and ectopic mouth tissue (Bottom). At 21 days RNAi, 5/7 animals display an anteriorly expanded dd_554 domain, and a 6/7 display posteriorly expanded NB.22.1e domain. At 40 days RNAi, all animals assayed display ectopic pharynges and mouth tissue. (F) Ectopic pharynges express the transcription factor foxA, dd_554, and are connected to the (6G10+) muscularized gut by an NB.22.1e+ esophagus. 6G10 is a muscle antibody, other markers are RNA probes. (G) activin-2 expression at 0 and 24 hours post amputation in a midbody piece. (H) t-SNE representation of clustered cells (dots) colored according to single gene expression or cluster assignment based on global gene expression. (Top left) 50,562 cells [50] obtained by Drop-seq are colored according to major planarian tissue type cluster assignment. (Top right) Each cell is colored and sized by the average normalized expression of activin-2. (Bottom) Muscle cells obtained by Smart-seq2 [51] are colored according to planarian [file pgen.1009466.s002.pdf]

Supplemental Figure 3

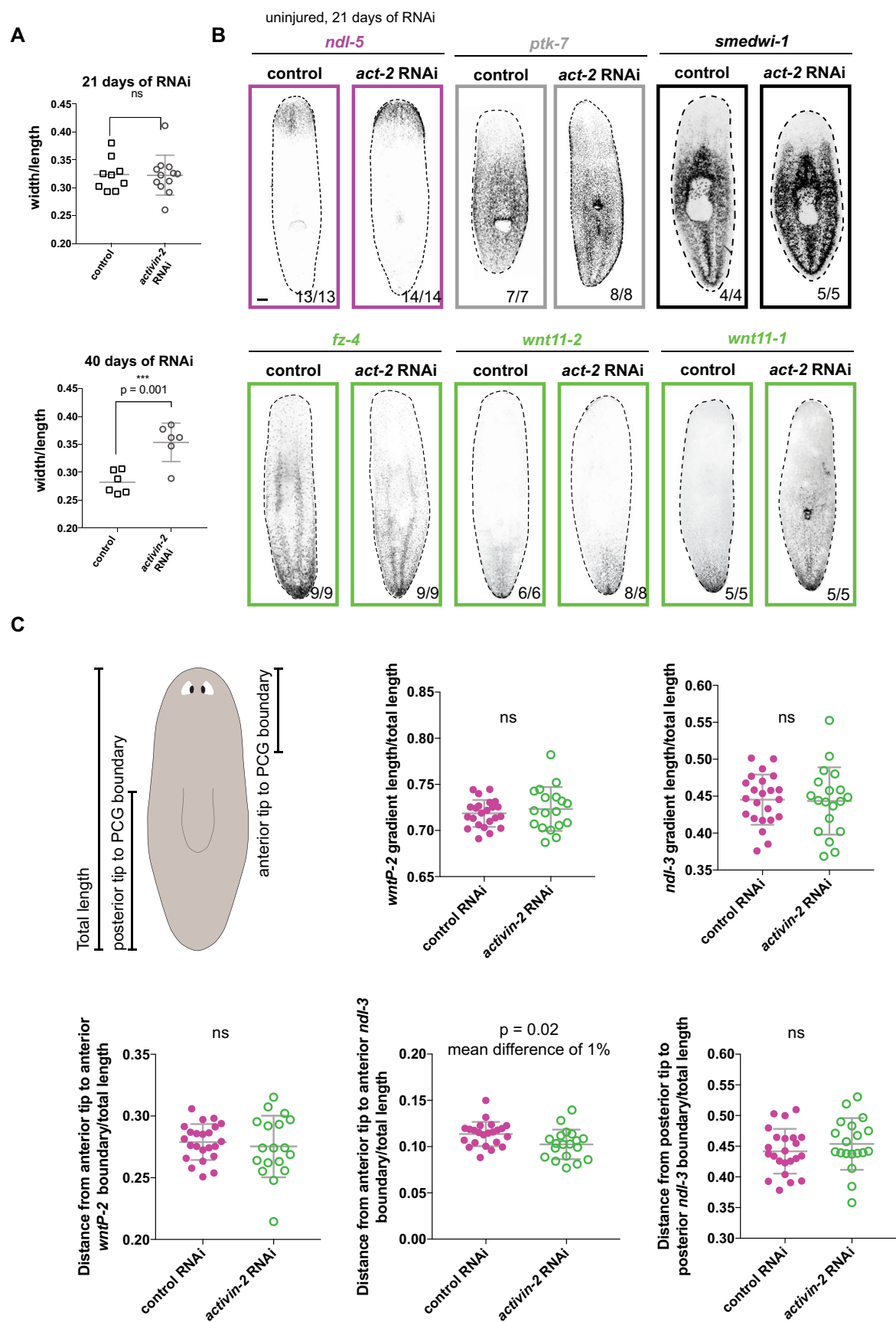

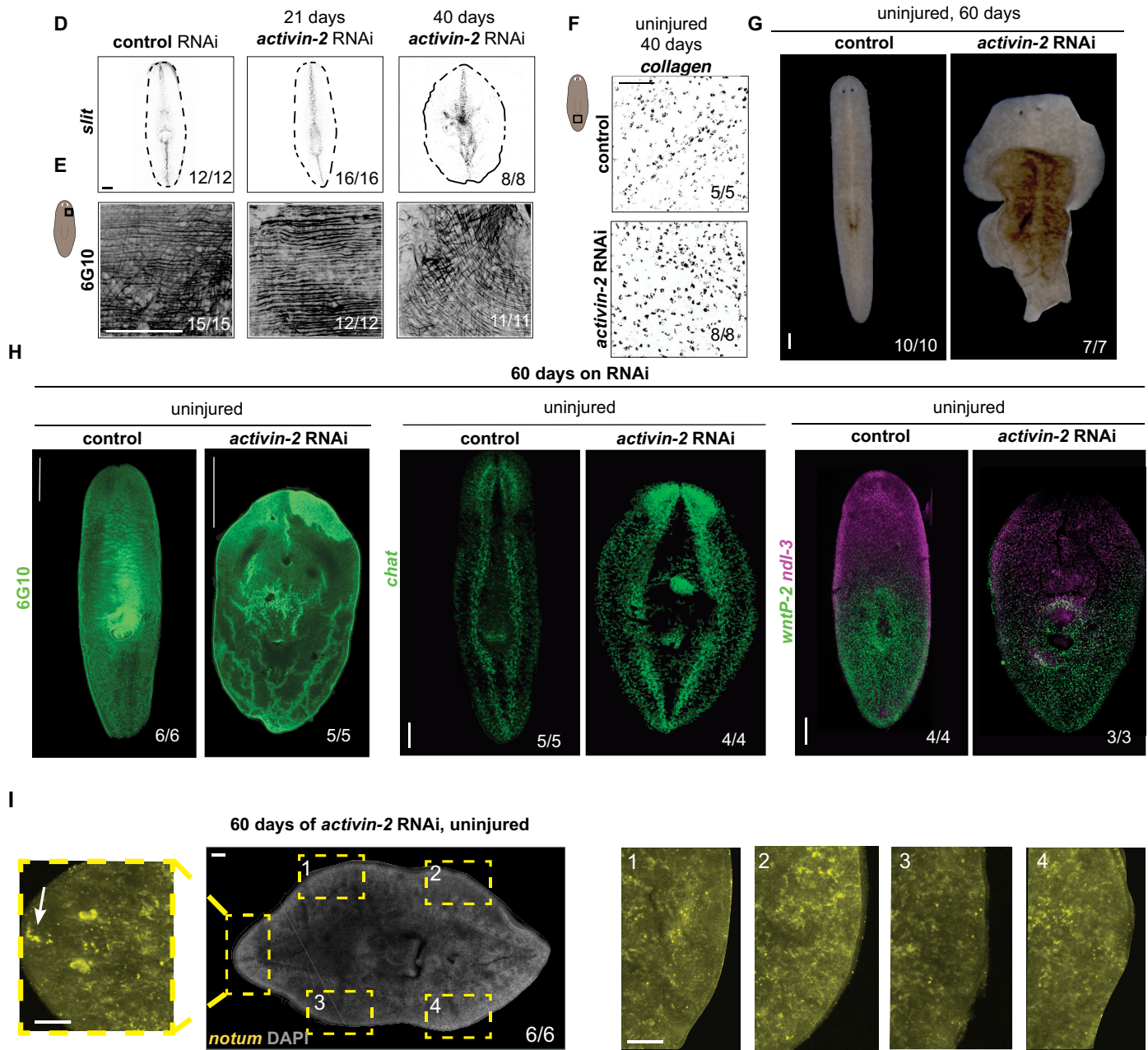

Supplement: S3 Fig — Related to Fig 2. (A) Graphs show body width/length per animal at different time points. Width quantified at widest point of the animal. (B) Intact activin-2 RNAi animals display proper PCG expression and pole presence at 21 days of activin-2 RNAi along the AP axis by FISH. (Pink) Anterior restricted genes, (Green) posterior restricted genes. act-2 is abbreviation for activin-2. (C) Quantification of PCG domain expression relative to animal length. The length to body ratio for ndl-3 and wntP-2 was blind scored following the landmarks show in the top left. Data are presented as mean +/- SD. A student’s t-test was performed to determine significance with a cutoff of p = 0.05. (D) Intact activin-2 RNAi animal midline (slit+) gene expression at 21 days and 40 days by FISH. (E) Intact animal muscle fibres (6G10+) at 21 days and 40 days activin-2 RNAi by immunofluorescence. 40 day RNAi animals display loss of orthogonal directionality of muscle fibres. (F) Intact activin-2 animal muscle cell gene expression (collagen-2) at 40 days of activin-2 RNAi by FISH. (G) Live images of intact control and activin-2 animals at 60 days of RNAi. (H) (Left) Immunofluorescence (6G10) of control and activin-2 animals showing muscle fibers, (Middle, Right) FISH of control and activin-2 RNAi animals showing central nervous system (chat), and PCGs (ndl-3, wntP-2) at 60 days of RNAi. (I) Intact activin-2 RNAi animals display maintenance of restriction of the anterior pole maker notum at 60 days RNAi by FISH. (PDF) [file pgen.1009466.s003.pdf]

**Supplemental Figure 4**

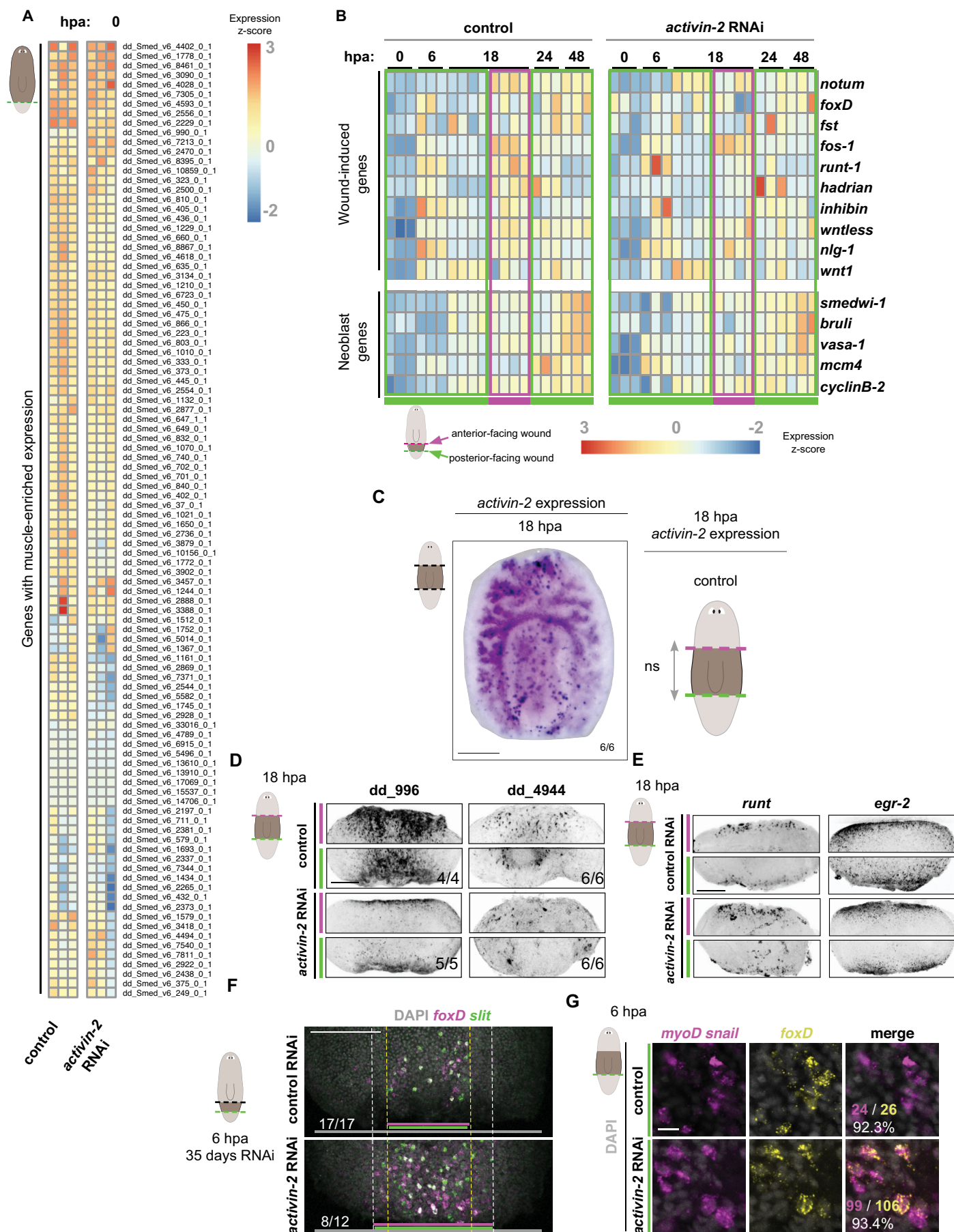

Supplement: S4 Fig — Related to Fig 3. (A) Heatmap of top 100 muscle specific genes annotated in [19] from bulk sequencing of posterior-facing wounds at 0 hours post amputation after 21 days of either activin-2 or control RNAi (three wound sites per replicate). Each gene is a row, and each replicate is a column. Related to S2 Data. (B) Heatmap of wound induced gene expression from bulk sequencing of posterior-facing wounds at 0, 6, 18, 24, and 48 hours post amputation, and anterior-facing wounds at 18 hours post amputation (three to four wound sites per replicate). Each gene is a row, and each replicate is a column. Related to S2–S4 Data. Top: known wound induced genes expressed in muscle (notum, foxD, fst, inhibin-1, wntless, nlg-1, wnt1), epidermis (hadrian), neoblasts (runt-1), and broadly induced (fos-1). Bottom: known neoblast genes, expression increases indicate an increase in cycling cell number (C) Left: in situ hybridization of activin-2 at 18 hpa, Right: carton showing DE-Seq result from bulk sequencing data that there is no significant difference in activin-2 expression at 18 hpa between anterior- and posterior- facing wounds in control animals. (D-E) Wound-induced gene expression at 18 hours post amputation. Magenta denotes anterior-facing wound, and green denotes posterior-facing wound. (D) FISH shows genes that are expected to change given DEseq data. (F) Wound induced foxD expression is correlated to midline (slit+) width. activin-2 RNAi animals have a marked increase in midline (slit+) width by 35 days, and foxD expression at 6 hours post amputation is increased to a similar width (ventral view). (G) foxD expression in activin-2 RNAi animals is specific to longitudinal muscle (myoD+, snail+) at posterior-facing wounds by FISH. Pink number is number of myoD/snail+ cells co-localized with foxD. Yellow number is total foxD+ cells. White number is percent foxD cells that are longitudinal fibers. Scale bars represent 200 μm, except for the high magnification panel in S4G wher [file pgen.1009466.s004.pdf]

Supplemental Figure 5

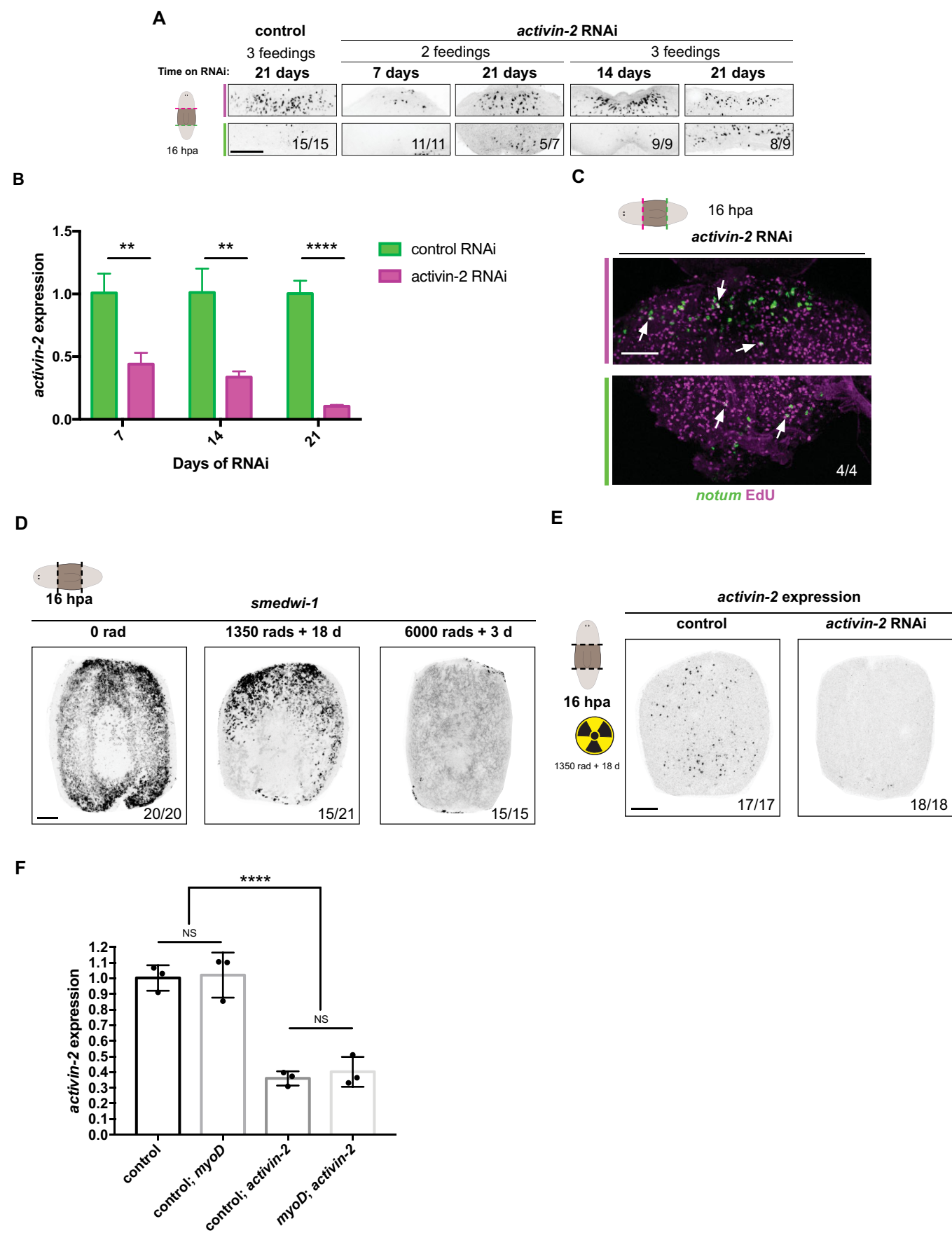

Supplement: S5 Fig — Related to Fig 5. (A) Wound-induced notum expression at posterior-facing wounds first appears after 21 days of RNAi (18 hpa) by FISH. (B) RT-PCR quantification of activin-2 expression at 7, 14, and 21 days of activin-2 or control RNAi. Relative expression is plotted as 2-ΔΔCT values. Data are plotted as mean ± S.D. NS if p>0.05. (C) Zoom out of EdU labeling with notum FISH seen in Fig 5A. Anterior- and posterior-facing wounds of activin-2 RNAi are shown. Animals were fed with EdU once at day 15 of activin-2 RNAi. (D) smedwi-1 (neoblast marker) gene expression in different irradiation conditions performed in Fig 5B and 5C. Left: control with no irradiation, Middle: 18 days post 1350 rads, Right: three days post 6000 rads. (E) activin-2 gene expression across conditions performed in Fig 4D and 4E. (F) RT-PCR for of activin-2 for RNAi condition stated. Relative expression is plotted as 2-ΔΔCT values. Data are plotted as mean ± S.D. NS if p>0.05. Magenta denotes anterior-facing wound, and green denotes posterior-facing wound. Scale bars represent 200 μm. (PDF) [file pgen.1009466.s005.pdf]

## Supplemental Figure 6

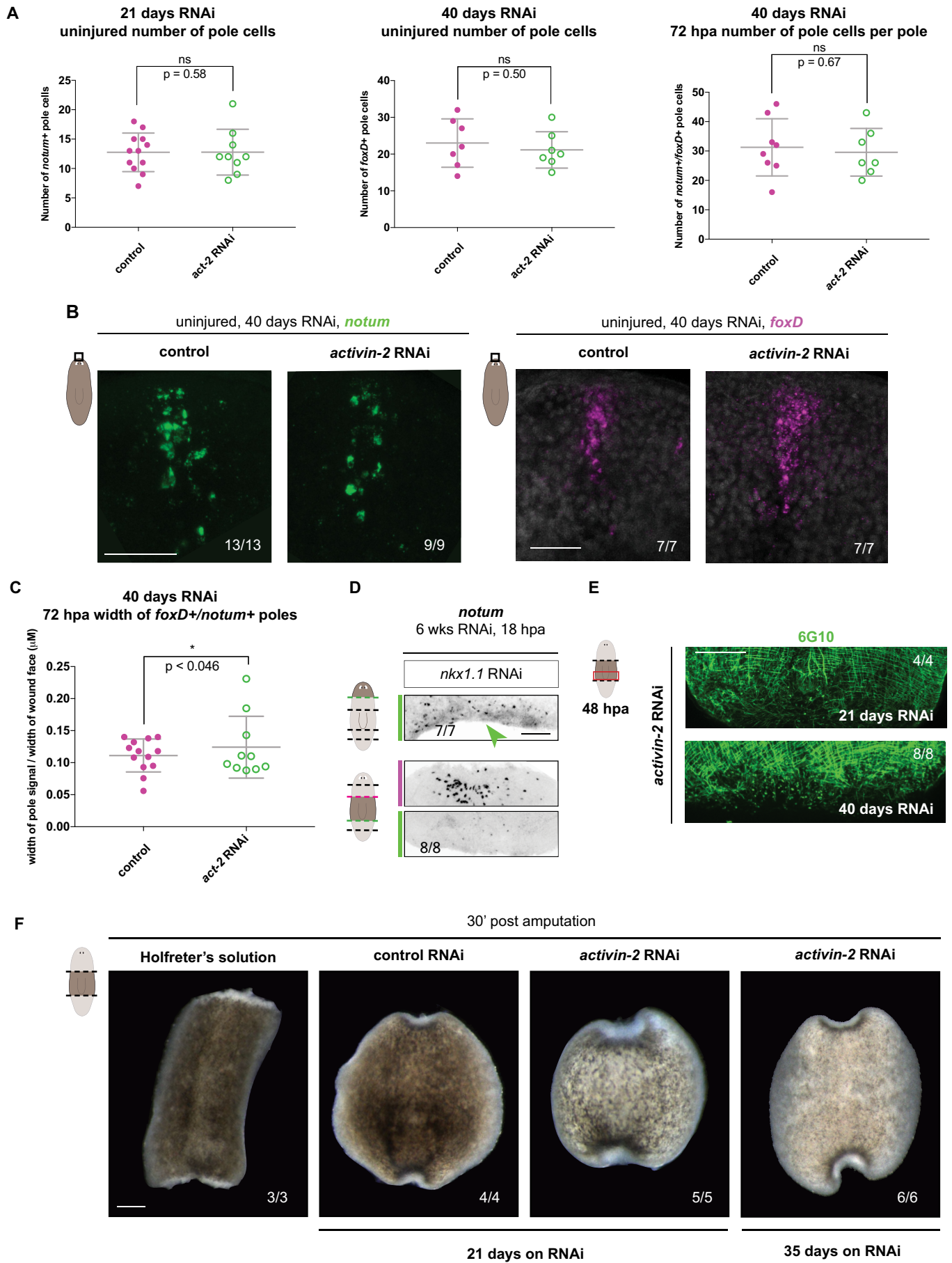

Supplement: S6 Fig — Related to Fig 6. (A) Number of pole cells (notum+ and/or foxD+) of (Left) intact animals at 21 days RNAi, (Middle) intact animals at 40 days RNAi, and 72 hpa (hours post amputation) animals at 40 days of RNAi. Student’s t-test used to determine statistical significance. NS is >p 0.05 act-2 = activin-2. (B) FISH of poles (notum+ or foxD+) of intact animals at 40 days RNAi. (C) Quantification for Fig 6A. Width of control and activin-2 RNAi animal poles (40 days of RNAi) at 72 hpa. Each datapoint is width of pole/width of wound face. NS >0.05. (D) notum expression at wounds in a head fragment and a midbody fragment after six weeks of nkx1.1 (circular fiber transcription factor) RNAi. Green arrow shows elevation of posterior-facing notum at 18 hours post amputation. (E) Immunofluorescence (6G10) showing muscle fibers at posterior-facing wounds at 48 hpa. (F) Live images of animals showing wound contraction at 30’ post amputation. Holtfreter’s solution inhibits muscle contraction, and was used as a negative control. (PDF) [file pgen.1009466.s006.pdf]

Supplemental Figure 7

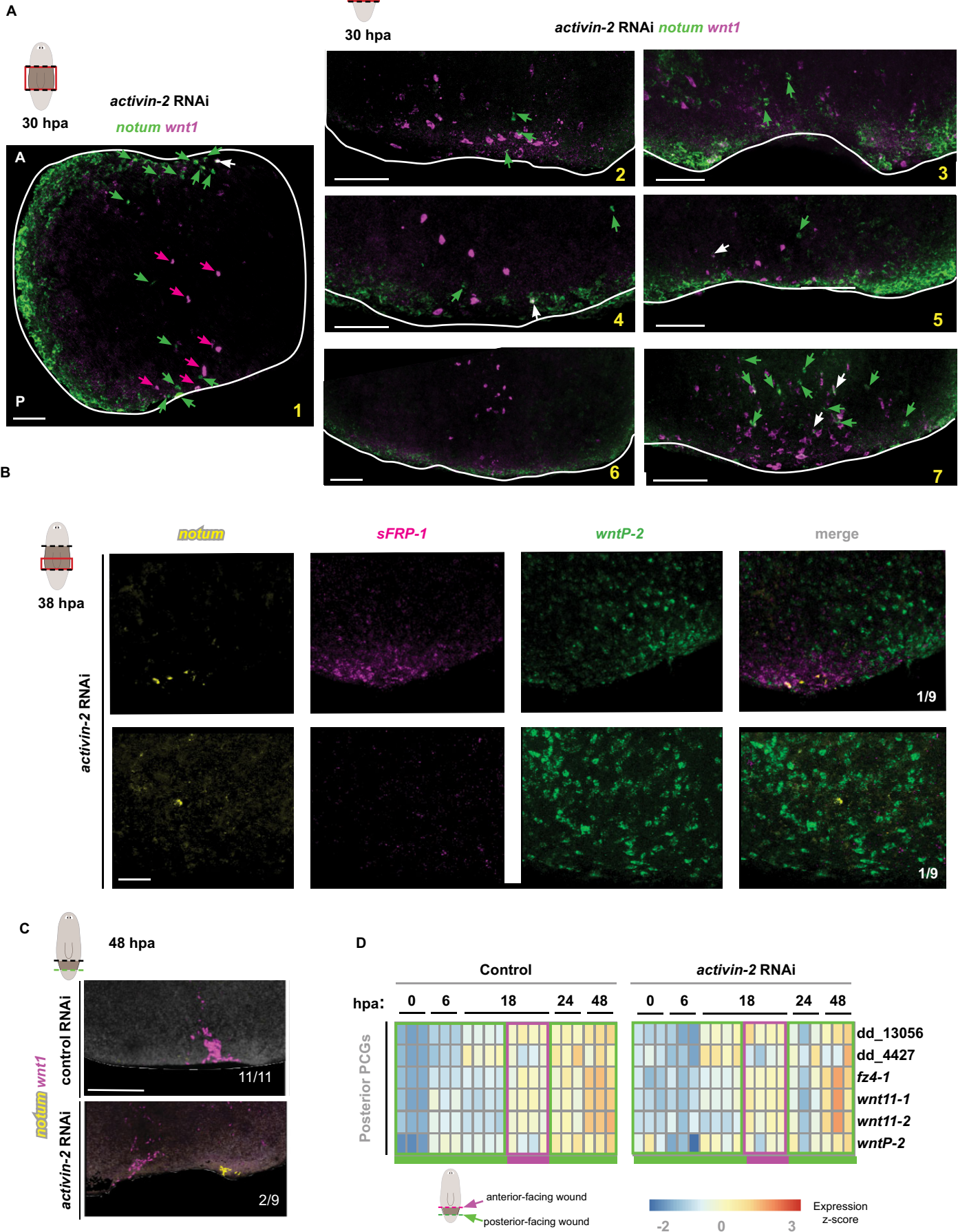

E

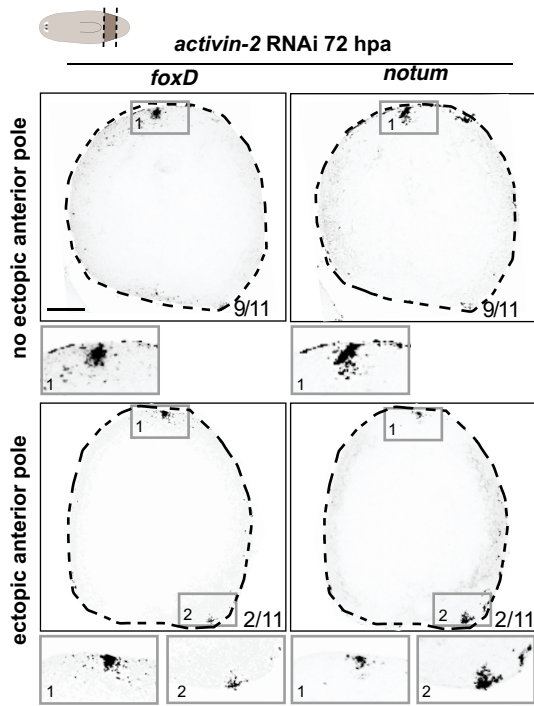

F

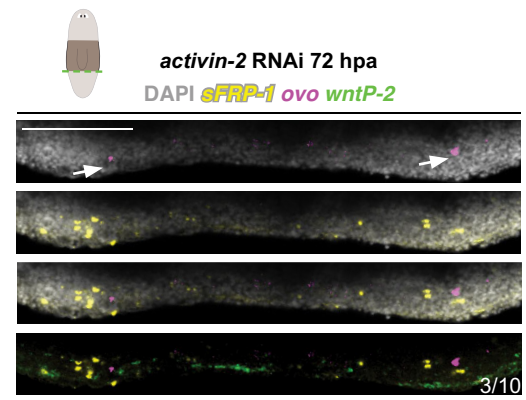

Supplement: S7 Fig — Related to Fig 6. (A) FISH shows expression of anterior pole marker (notum), and posterior pole marker (wnt1) in the same posterior-facing wound (Left: also shows anterior-facing wound) of an activin-2 RNAi regenerating fragment at 30 hours post amputation. Left: Magenta arrows show wnt1 cells proximal to posterior-facing wound. Green arrows show notum+ cells. White arrows denote cells that co-express notum and wnt1. 19 total animals were examined with varying FISH patterns; representative images of this experiment are shown here and in 7C. (B) FISH shows expression of anterior pole marker (notum), anterior PCG (sFRP-1), and posterior PCG (wntP-2) in the same posterior-facing wound (B). (C) FISH shows expression of anterior pole marker (notum), and posterior pole marker (wnt1) in the same posterior-facing wound of an activin-2 RNAi regenerating fragment at 48 hours. (D) Heatmap shows posterior PCGs and markers from bulk sequencing of posterior-facing wounds at 0, 6, 18, 24, and 48 hours post amputation, and anterior-facing wounds at 18 hours post amputation. Each gene is a row, and each replicate is a column. Related to S2 Data. (E) foxD and notum are co-expressed in the ectopic anterior pole at the posterior-facing wound of an activin-2 RNAi animal fragment at 72 hours post amputation. (F) Lower magnification of image shown in Fig 7C denotes close proximity between ovo+ eye progenitors and the anterior PCG sFRP-1 at a posterior-facing wound of an activin-2 RNAi animal at 48 hours post amputation. Scale bars, 200 μm. (PDF) [file pgen.1009466.s007.pdf]
