# Supplementary material for: activin-2 is required for regeneration of polarity on the planarian anterior-posterior axis
Source: PLoS Genet. 2021 Mar 29;17(3):e1009466. doi: 10.1371/journal.pgen.1009466 (PMC8057570; doi:10.1371/journal.pgen.1009466)
Supplement: Attachment — Submitted filename: Cloutier_Reply to reviewers.pdf [file pgen.1009466.s012.pdf]

Cloutier et al. Reply to Reviewer comments

We thank the reviewers for their thoughtful comments to improve the manuscript. Please find our responses to each point below **in bold text**.

Reviewer #1

In this manuscript, Cloutier and colleagues investigate the mechanism through which planarian Activin-2 affects body polarity and regeneration. Building upon prior work from the Reddien group and others, this manuscript shows that activin-2 RNAi causes pleiotropic phenotypes that sometimes include ectopic heads in the posterior of the animal. The authors further show evidence that notum expression is not asymmetrical at 18 hours post-amputation in activin-2 RNAi animals, which may lead to subsequent polarity defects. This work is thorough, with well-designed experiments and beautiful images and thorough quantification throughout. The work adds to our understanding of planarian polarity signaling, particularly after injury. The authors present a strong body of work that should be interesting to the readers of PLoS Genetics. The manuscript should be acceptable for publication once the following concerns are addressed. Nearly all of the listed concerns should be addressable with writing changes or potentially quantification or inclusion of existing results.

Major concerns:
1. At times, the authors misrepresent the novelty of their contribution in this manuscript. For example, they make claims like "Activin signaling was previously not known to regulate regeneration polarity" and "Here we report an unexpected role for Activin signaling in controlling head-versus-tail regeneration in planarians." They also declare that they are showing a "novel" activin-2 RNAi phenotype. These claims are somewhat misleading. Activin-2 has been previously characterized in Gavino, et al (eLife 2013) and Roberts-Galbraith and Newmark (PNAS 2013) (activin-2=activin in the PNAS paper). Undoubtedly, this new manuscript reports additional phenotypes (ectopic posterior heads), probably due to better penetrance of the RNAi or differences in experimental timing. Additionally, this manuscript reports a more detailed mechanism for this phenotype and takes the prior findings in exciting new directions. But throughout the manuscript, the authors should be clearer about what is new in this work and how it relates to prior research. In particular, the data presented in the PNAS paper implicated Activin signaling in polarity and in inhibition of anterior fates, so this manuscript ties nicely into the prior model.

We have added more description on previous work on *follistatin* and *activin-2* in the intro. This background describes the impact of *follistatin* RNAi on anterior regeneration and that this has been found to be explained by causing increased *wnt1* expression early in the wound response *(Lines 97-117).* Line 120: We added the text: "through regulation of asymmetric *notum*" to describe how the phenotype described here was unexpected compared with prior work. Line 519: We added "at posterior-facing wounds" to describe more specifically what was unknown about Activin signaling regulating polarity. We also highlighted in the discussion the ways in which the *activin-2* phenotype is both different and more specific to wound-based polarity (*notum* expression asymmetry) than the *follistatin* phenotype. We hope that these changes give a better context for this work – both crediting previous work on *follistatin* and *activin* in regeneration and explaining that factors contributing to *notum* based polarity at wounds were previously unknown (Lines 503-520).

2. The data that are used to support the argument that Activin-2 is important in Notum asymmetry are sometimes a bit unclear.
A) First, the data presented only support symmetrical notum expression after activin-2 RNAi at 18h post-amputation (Supp. Fig. 4A). Is that correct? I think that the average reader might reasonably conclude that Notum expression is symmetrical throughout regeneration after activin 2 RNAi (e.g. "We conclude that Activin has an essential role in the asymmetric activation of notum… during planarian whole-body regeneration."). The authors should consider moving the data from Supp. 4A into the main text to avoid this confusion, perhaps with more quantification of the symmetry of expression over time. Words like "transient" to remind the reader of the temporal nature of the

phenotype might also be helpful.
B) The data in 3B are also a bit confusing, perhaps because Notum is expressed predominantly in the anterior (normally) and thus posterior RPM is hard to interpret in context. Are data available to show both posterior and anterior RPM (and/or potentially the anterior:posterior ratio) over time?

C) It seems that there is a bit of a logical disconnect between the transience of the symmetrical Notum phenotype and the striking nature of the long-term phenotype (including re-expression of posterior Notum in posterior heads, Fig. 6B). If Notum expression (or rather the symmetry of Notum expression) is back to normal by 24 h post-amputation, how do the authors think that the longer-term phenotypes arise? I think this disconnect could probably be addressed in the text or even in the discussion to help a reader connect the different elements of the phenotype in one coherent model. The model figure (Fig. 7) seems to omit a complexity of the story if polarity is symmetrical at 24 hpa.

A) **We moved figure S4A into the main text (now Fig 4A), and added the description of "occurred by 18 hpa, but was not present at early timepoints" to the expression of symmetric WI *notum* along with further temporal description (Lines 286-288). Furthermore, the RNA-sequencing data presented in Fig 3B demonstrated that *notum* expression at posterior-facing wounds was not elevated until between 6 and 18h compared with control animals.**

B) **The timecourse data is only available for posterior-facing wounds. Bulk sequencing was performed at anterior-facing wounds only at 18 hpa to compare to the levels at posterior-facing wounds (shown in the left side of panel B). At the 18hpa timepoint there was no significant difference between anterior-facing wounds and *activin-2* RNAi posterior-facing wounds. We moved the "posterior-facing wounds" label to the graph in 3B to the top, and made it bigger to make that attribute clearer.**

C) **We thank the reviewer for the suggestion to add to the discussion to clarify this point for the reader. The first important point in considering this is that there are two phases of *notum* expression involving different cells - the wound-induced phase (in existing muscle) and the later expression in the anterior pole (which are derived from neoblast progenitors). *notum* wound-induced expression normally declines at anterior-facing wounds by ~24h, presumably when a burst of wound signal wanes. For example, see current Figure 4A and Petersen et al. 2011. About a day later, a new pole is visible (precise timing can vary). During this interim time, anterior-PCG expression (e.g., *sFRP-1, ndl-5*) initiates. This suggests that wound-induced *notum* can activate a head program of anterior PCG expression and posterior PCG reduction that allows neoblasts to become specified into pole progenitors that make an anterior pole. The anterior pole is then required for maintaining and further subdividing this shifted PCG environment. Therefore, we view wound-induced *notum* expression declining by 24h in existing muscle as similar to the normal head regeneration program. (Note also, that measured here and in normal anterior regeneration is wound-induced *notum* mRNA, and its protein product might perdure longer). Note also that the phenotype at posterior-facing wounds only progresses to anterior pole formation and an ectopic head some of the time, and together with a tail forming - this could also lead to somewhat lower *notum* at posterior-facing wounds of *activin-2* RNAi animals during the initial period of pole progenitor specification than at normal anterior-facing wounds. We have added more description to the text to clarify this aspect of the model and of normal anterior regeneration (Lines 374-421); we also added more experimental detail regarding the timing of anterior-PCG**

expression, reduction of *wntP-2* expression, and ectopic anterior pole nucleation at *activin-2* RNAi posterior-facing wounds (Fig 6 and Fig S7A,B,E).

3. The authors argue that the activin-2 phenotype with regards to polarity is “regeneration-specific.” However, the authors also state that “subtle anterior shifting of wntP-2 expression domain length could not be excluded.” Have the authors attempted to quantify the expression domains for ndl-3, wntP-2, and ndl-5 (e.g. head to anterior margin of the domain, length of the domain versus length of the animal) at day 20 post-amputation? The images do look somewhat different, which might mean there is also a homeostatic phenotype. Have the authors looked at AP gradient markers at later time points (e.g. 6 weeks). By that time point, there is a dramatic change in animal shape as well as a change in slit expression (Supp. Fig. 2) which indicates that ML polarity is affected homeostatically. My expectation is that AP might be affected then more strongly than at 3 weeks/20 days. If AP effects are not exclusive to regeneration, the language around this point probably need to be altered.

We now labeled many more animals with *wntP-2* and *ndl-3* RNA probes to address this question.

We blind scored animals and quantified the normalized gradient length of both PCGs, as well as their normalized boundaries. We used the anterior boundary of *ndl-3* as a proxy for the head-midbody boundary, and the posterior boundary of *ndl-3/*boundary of *wntP-2* as a proxy for the midbody-tail boundary.

We did not see a significant difference in any of these comparisons, except for a significant though small shift of the *ndl-3* anterior boundary (the anterior boundary shift distance reflected ~1% of total animal length). We have added this data to the manuscript as Figure S3C.

Minor concerns:
1. The authors show that activin-2 RNAi causes muscle disorganization and slit misexpression. The Activin pathway (at least the receptor) has also been shown to have a role in fissioning behavior (Arnold, 2019). Did the authors note any changes in behavior/movement in the activin 2 RNAi animals? Is it possible that muscle function is perturbed in these animals and – if so – could wound closure be affected? If failed would closure results in a wider or more uneven starting point for regeneration, could this contribute to splitting of heads/tails?

This is an interesting idea, and we appreciate the suggestion.

We performed live imaging of animals after wounding to assess wound contraction and closure, which assessed by the presence of a dark band of contracted tissue at wounds. Animals exposed to Holtfreter’s solution fail to form this contraction band, with apparent open wounds. By contrast, we did see ~normal contraction present in *activin-2* RNAi animals at 21 days RNAi and at 35 days RNAi. We have incorporated these data as Figure S6F.

We also performed a muscle antibody staining (6G10) at 48 hpa on 21 day and 35 day RNAi animals to assess wound closure and to observe intact muscle at wounds. The data are incorporated into the manuscript as a part of supplemental Figure S6E.

We are hesitant to comment on behavior in the manuscript as we have not focused on that over the course of this project. However, we have noted that *activin-2* RNAi animals rarely fission.

2. Did the authors try to “rescue” split heads or tails with slit(RNAi) to determine if the slit domain expansion was causative in other phenotypes?

We tried this experiment, but found the outcome to be difficult to interpret. Concerns with the experiment include: 1) variability in number of heads split/ectopic posterior heads, 2) overall low penetrance of the phenotype we are trying to suppress that could be potentially weaker when feeding 2 dsRNA constructs throughout, 3) even if we saw suppression, this could just be “step wise” epistasis (blocking midline formation might preclude capacity for ectopic head splitting regardless of the mechanism at play). We therefore decided not to include the results in this manuscript.

3. In Fig. 1B, there seems to be extra chat staining in the anterior of the animal (in the middle of the head). Is this often seen in these animals? Do the authors think that this is out of place pharyngeal tissue, brain tissue, or something else? This might be another part of the improved phenotype worth mentioning.

We evaluated this structure using DAPI and it appears to be consistent with ectopic pharyngeal tissue. We examined other animals as well and added our DAPI analysis of this structure to the supplement as part of Figure S1D.

4. The authors do not show expression of activin-2 after pre/postpharyngeal amputation and 18hpa. Is there evidence that activin-2 expression is asymmetric at 18 h? I think this information would be helpful in imagining how activin-2 might affect notum, but I would not recommend holding up the paper for this experiment, given current lab shutdowns for COVID-19.

We performed ISH at 18 hpa on trunks and added it to Fig S4B (moved from S1E). We do not see any asymmetry of expression of *activin-2* at 18 hpa in WT animals. Since this ISH used NBT-BCIP, we have added that protocol to the methods section.

We have also assessed the control animal RNA seq data at 18 hpa, and did not see any asymmetry of expression. A cartoon depicting this sequencing analysis has been added to the Fig S4B.

5. Can the authors please clarify in the figure legend which animals were used for quantification in Fig. 1C? The denominators are not the same, so I think some stained animals were used for some but not all quantification, but I can’t be sure.

These represent two separate sets of animals. The pharynges were assessed in intact animals by DAPI staining, while the other components of the phenotype were assessed in a separate set of animals that had regenerated from a transverse amputation at 14 days post amputation.

We sought to clarify this by adding more labels to the visualization.

6. Is pigmentation affected by long-term activin-2 RNAi (Fig. 2A)?

We also noted this possibility, but are uncertain how to interpret the finding given that the animals have changed anatomy and body proportions in some cases. Further comment on this would require more study to see if any aspect of the pigment lineage is affected. However, no white patches were observed, as is seen with other pigment lineage defects.

7. Can the authors clarify the dosage of dsRNA in the methods (concentration or total mass)? Given that this work shows new phenotypes, potentially due to RNAi effectiveness, dosage information would be helpful.

Concentrations were between 5-8 ug/ul for each prep of dsRNA. This is described in the methods.

8. The data from irradiation experiments were a bit challenging to interpret, especially since the time points post-irradiation are not 18 hpa. The result in Fig. 5B indicates that the asymmetry phenotype is stem cell independent. But the sub-lethal irradiation experiment with a time frame in which stem cells are largely recovered (Supp. 5C) shows no symmetric expression. Is the argument that sublethal irradiation prevents activin 2 RNAi animals from misspecifying muscle (or accumulating disorganized muscle) and then without muscle disorganization you don't see symmetric notum expression? I think that, particularly for non-expert readers, the take-home message for these experiments could be clarified.

Thank you for finding this typo. We performed irradiation experiments at both 18 and 16 hpa, and the findings were similar – however the data presented is for 18 hpa. We fixed this in the figure.

Thank you for also asking for clarification. Taken together, our hypothesis is that *activin-2* is required for specifying 'polarity competent' muscle because when tissue turnover is perturbed by sub-lethal irradiation symmetric wound *notum* is suppressed.

We also added a new experiment in which we inhibited new muscle production with *myoD* RNAi. *myoD* RNAi blocks production of new longitudinal muscle cells, where *notum* is expressed. This also suppressed the *activin-2* RNAi phenotype, consistent with the irradiation experiment, and further supporting the conclusion that muscle fiber turnover is required for the *activin-2* RNAi phenotype. We added to the text to clarify the results (Lines 319-344) and expanded upon this point in the discussion.

9. For RNAi experiments in which an interesting phenotype is seen in a minority of animals (e.g. Fig. 1B, 6C, 6E), it would be helpful to include the phenotypes that are most prominent, as well. This will help the reader to interpret data properly and get a feel for the full range of RNAi phenotypes.

We agree this will clarify the data and make it easier to interpret. We added example images for animals that did not display polarity reversal (Fig S2C), but did display ectopic mouth tissue. We showed some of the variation that exists for 6C/E in S7B at 38h, and in the other animals we did not see distinction from control.

Reviewer #2

In the manuscript by Cloutier et al., the authors investigate the roles of activin-2 in establishing regeneration polarity upon regeneration in the planarian S. mediterranea. Following transverse amputation of a planarian, a regeneration polarity decision must be made to appropriately regenerate a head at anterior-facing wounds and a tail at posterior-facing wounds. The expression of notum, a wnt signaling inhibitor, is the first indication of a differentiation between the anterior and posterior wound sites, as notum is preferentially expressed at anterior-facing wounds. This work demonstrates that following knockdown of activin-2, amputated worms regenerate ectopic heads at posterior facing wounds. Activin-2 knockdown worms also experience axis bifurcations at anterior and posterior regenerating blastemas. RNA sequencing analysis shows that activin-2 knockdown worms have symmetric notum expression at anterior and posterior wounds, but no other functionally significant alterations in early wound response gene expression. Interestingly, the authors show that production of new longitudinal muscle cells is required for this symmetric notum expression, suggesting that activin-2 exerts its effects during muscle cell differentiation. Later in regeneration, activin-2 RNAi worms express both anterior and posterior positional control genes at posterior-facing wounds, resulting in the generation of discrete anterior structures in the posterior. This suggests that activin-2 restricts wound-induced notum expression to anterior-facing wounds to promote tail regeneration at posterior-facing wounds and that activin-2 is a regulator of regeneration polarity. This work identifies the first regulator of asymmetric notum activation, providing important insight into the question of how planarians differentiate between anterior and posterior wounds. The data are high quality, however, some key experiments are missing, and/or over-interpreted. While the phenotypes are of interest, the mechanism for the most interesting phenotype, the axis bifurcation, is not thoroughly investigated.

Major Concerns

1. In the Introduction, a summary of known follistatin and activin and TGFB phenotypes known in planarians so far is warranted (which is significant). The current lines about activin and follistatin in planarians (lines 103-107) are vague and not helpful to the reader put your study into context of what is known and what is missing.

We agree with this suggestion and have re-worked the introduction to help the reader understand this background on *follistatin* and *activin* more fully *(Lines 97-117, 503-520)*.

2. Need proper phylogenetic analyses of the TGFB family to resolve whether planarian activins are activins or myostatins in order to resolve exactly the issues raised in lines 122-128.

We performed Bayesian phylogenetic analyses, which indicates that Smed-Activin-2 is in a clade with Activin proteins in other organisms. However, this clade also contains a Myostatin-like protein and therefore do not want to exclude the possibility that *Smed-activin-2* could be derived from an ancestral, related, *Myostatin-like* gene (which also would interact with Follistatin and signal through Smad2/3). In Mus musculus genes that contribute to Activin proteins are called *inhibin* when not dimerized, we have used this nomenclature in the tree. We added the phylogenetic analysis as Supplemental Figure 1, and discuss it in the text.

3. Line 168: seems like an over-interpretation to fit the authors "story" as opposed to objectively stating the reality that activin-2 is detected in every major cluster in scRNAseq, and in muscle

subclustering, high expression was seen in DV-like and a sub-cluster of circular (not in all circular muscles as described and annotated in S1F).

The Drop-seq dataset visualization was modified as in Cote et al 2019, because of overall low abundance of *activin-2* expression in the dataset. We see how this could appear that *activin-2* may appear abundantly expressed in each cluster, when it is more that *activin-2* is only lowly expressed in this dataset, and have replaced that plot with the Digiworm output. The two plots are shown below for comparison – with the Digiworm-based plot on the right.

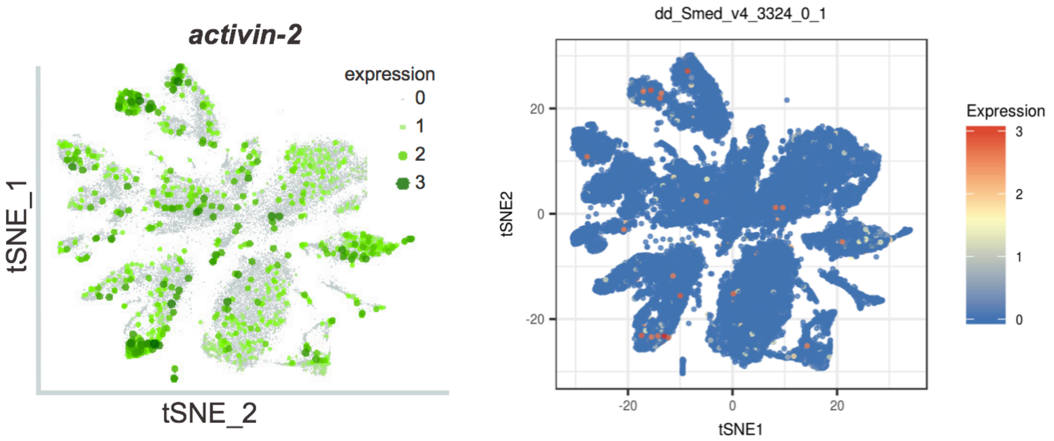

The Smart-seq2 DV-like cluster is not well characterized in Scimone et al 2018, so we are hesitant to claim anything about the identity of cells in that cluster. However, we agree that *activin-2* expression is not limited to *nkx1-1+* cells in the muscle cell data and modified the text to reflect this. *activin-2* is expressed in several tissues by FISH (pharynx, muscle, intestine) and we highlight that in the text (Line 185-186).

4. The role of symmetric notum expression in the regeneration of ectopic posterior heads is compelling. However, the association between symmetric notum activation and axis bifurcation during regeneration is still unclear. The model figure as well as the nkx1.1 experiments seem to suggest that axis bifurcation in both anterior and posterior blastemas occurs as a result of symmetric notum activation, but as notum activation in the anterior is normal in activin-2 knockdown worms, it is unclear how bifurcated blastemas form in the anterior. Do the sublethally irradiated worms from Fig 5C eventually regenerate? If so, do the activin-2 knockdown worms with asymmetric notum activation still develop bifurcated blastemas?

We do not think that the head anterior bifurcation is likely caused by symmetric *notum* activation at wounds, but likely reflects another role of Activin. The *nkx1.1* experiment was intended to see if *nkx1.1* RNAi lowers *activin-2* expression substantially enough to see any increase in posterior notum, which we did see observe in head fragments – although these heads did not form ectopic posterior heads. Unfortunately, most of the sublethally irradiated worms did not survive amputation so we were not able to assess them as a cohort during regeneration.

We modified the text to make it clearer that anterior head splitting is likely a consequence of a different mechanism than *notum* wound expression; we hypothesize this may be connected to nucleation of two anterior poles, with anterior bifurcation substantially increasing from 21 days to 35 days on RNAi when animals are widening, and potentially is associated with a defect in nucleating a single, focused pole (Lines 355-361). We separated this text section under its own header and described posterior-facing blastemas under a separate section header for clarity in the text.

5. There appears to be a larger number of cells expressing notum in uninjured activin-2 knockdown worms (Fig 2B) which was not discussed. This raises the question of whether there is a difference in the number of muscle cells expressing positional control genes during regeneration, and whether this contributes to the apparent increase in notum expression contributes to the bifurcations upon regeneration. In the cases where the anterior blastema regenerates as normal, is the number of pole cells normal as well? Quantification of foxD+ pole cells would address this issue across all phenotypes.

We performed additional experiments and added images and quantification to Figure S6 to address this question. We quantified the number of pole cells using *notum* and *foxD* as markers and there was not a significant difference in the number of cells that make up the pole in *activin* RNAi and control animals. Given this, we believe that any difference seen in Figure 2B is based on animal-to-animal variation.

We have however noticed in other parts of the paper that the *activin* RNAi animals have widened, including *slit*+ midline expansion. We therefore quantified poles in the blastema and see that these poles are wider on average at 40 days of RNAi. The visual impression of a widened pole, may give the appearance of an increased number of cells prior to counting.

6. This paper mentions that Follistatin is required for the missing tissue response and is a regulator of Activin, but did not explore the role of Follistatin in regulating activin-2 during regeneration. The authors report that the missing tissue response is normal in activin-2 knockdown worms based on the expression of neoblast genes in RNAseq (lines 222-227). This should be supported with quantification of proliferation during the first 2dpa. Additionally, if Follistatin does act to inhibit activin-2 as well as activin-1, double RNAi of follistatin with nkx1.1 could be used to test the hypothesis that the bifurcations seen in nkx1.1 knockdown worms are due to a decrease in activin-2 expression.

The outcome of *nkx1.1* and *follistatin* double RNAi is a little hard to predict. *activin-2* is downstream of *follistatin* and thus its perturbation is epistatic to *follistatin* perturbation. i.e., in a double *activin-2 ; follistatin* RNAi experiment, the *activin-2* phenotype is seen and not the *follistatin* phenotype. A similar prediction could be made for *nkx1.1* because it lowers *activin-2* levels. That said, *nkx1.1* does not completely eliminate *activin-2* expression, so it is possible that *follistatin* RNAi would lead to the remaining Activin-2 protein being produced being more active. However, to what degree is hard to predict, and we feel the experiment is therefore limited in potential. Regardless, we have tried double RNAi experiments with *nkx1.1*; *nkx1.1* RNAi results in a low penetrance of head splitting alone and when dsRNA was split to be half *nkx1.1* and half control dsRNA head splitting was not obtained in the sample size used. This technical challenge also makes this experiment impractical.

We quantified H3P levels at 72 hpa, and with the power of this experiment do not currently see a significant difference between control and *activin-2* RNAi. We decided not to include this result in the paper however, as we think the data could be trending that *activin-2* has increased mitoses; we think further experimentation may need to be done to explore the missing tissue response properly in the context of Activin signaling, which could be an interesting future avenue. The data obtained is presented below:

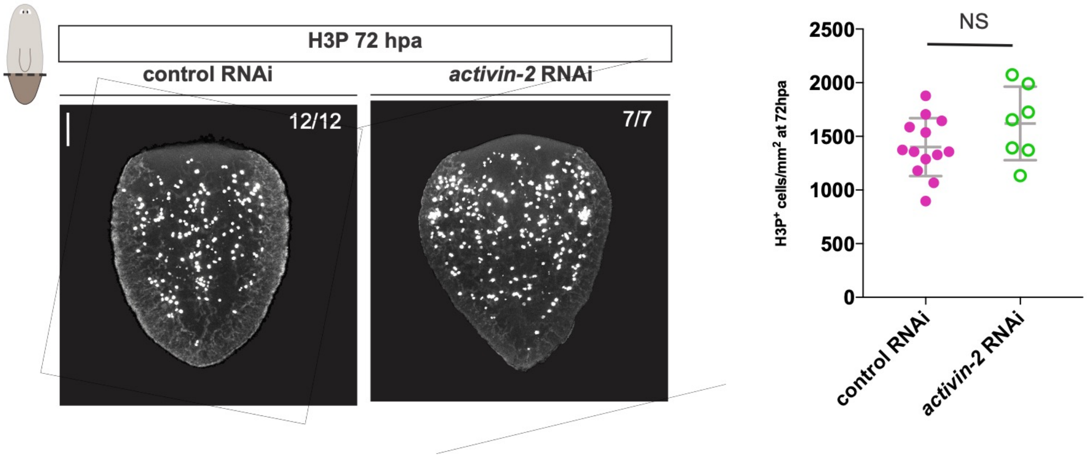

7. A more in-depth analysis of the muscle cell subsets involved in the activin-2/notum response to injury would be beneficial, particularly given the extensive work previously done by this group on muscle cell subsets. The claim that newly-formed longitudinal muscle fibers are responsible for asymmetric notum activation could be better supported using myoD knockdown worms, where differentiation of new longitudinal muscle fibers is blocked (assuming it is feasible to generate myoD/activin-2 RNAi worms).

Thank you for this excellent experimental suggestion. We performed the experiment, and saw that *myoD* RNAi did in fact suppress the *activin-2* RNAi phenotype. As expected, *myoD* RNAi decreased overall wound-induced *notum* expression as was shown in Scimone et al 2017. Interestingly, however, *myoD* RNAi depleted *notum* at posterior-facing wounds in *activin-2* RNAi animals at a much greater proportion. This is consistent with these *notum*+ cells at posterior-facing wounds being newly formed longitudinal fibers.

qPCR for *activin-2* was performed in this experiment to demonstrate that the effect was not simply caused by dilution of the dsRNA. We have incorporated these findings as Figure S5F and Figure 5D.

Minor Concerns

1. Structural issues (citing fig S3A before S1F). Never mentioning the top of S1F.

Thank you for noting this, we edited the figure callouts to reflect the correct order. We also comment on the data from prior S1F (now S2H) in the text that *activin-2* is not represented well enough in the Drop-seq data to make strong conclusions from this particular plot.

2. S1F would be helpful to be next to Fig 1D (or move 1D to supplemental).

Because *activin-2* is not well represented in the Drop-seq data we are hesitant to move S1F to the main figure, but we do think it is important to note that *activin-2* is not itself expressed in an anterior-posterior gradient, which is why we advocate for keeping prior 1D (now 1E) in the main figure.

3. The implications of the irradiation experiments were not fully discussed in the text. Do these results suggest that longitudinal muscle fibers have anterior/posterior orientation 'encoded' by activin signaling during differentiation? This is an interesting point that warrants further discussion.

Thank you for asking for clarification. What you have stated above is correct. Taken together, our hypothesis is that *activin-2* is required for specifying 'polarity competent' muscle since when tissue turnover is slowed by sub-lethal irradiation symmetric wound *notum* is suppressed.

We added to the text to clarify this in the results (Lines 334-341 added the *myoD* RNAi data that supports this, and expanded upon this point in the discussion (Lines 466-470).

4. 40 days of RNAi treatment was used to determine the effect of activin-2 knockdown at homeostasis. This ruled out other phenotypes excluding multiple pharynges in uninjured activin-2 knockdown worms. This time frame does not seem long enough to allow for sufficient tissue turnover.

We scored intact animals at 60 days of *activin-2* RNAi including live images. These animals became difficult to incorporate into certain analysis as they have significant change in shape with a high degree of variation, including irregular tissue shape that no longer preserves their 'flatness' making it difficult to quantify PCGs, and other domains using FISH. We do however have PCG stainings suggesting that *notum*, *ndl-3,* and *wntP-2* expression are maintained in the correct order in these animals, although gradient quantification is difficult due to the variability in dimensions of these animals.

This data has been incorporated into Figure S2G-I.

5. Figure 3C: the RNA sequencing experiments are conducted at different time points with tissue at different amputation sites. It was unclear why the different tissue fragments were analyzed, as this was not addressed in the text.

We added context in the text for fragment choice including specifying that 1) we were interested in the biology of posterior-facing wounds, as this is the site of ectopic head formation, 2) we knew that we would want to compare anterior to posterior at 18 hpa as we had seen ectopic *notum* expression at this time point (Lines 250-252).

We also changed the cartoons for Fig 3C to make this figure easier to read.

6. The authors state that some genes changing in RNAseq do not look different by FISH in Fig. 3D.

However, the images shown look substantially lower in the posteriors of activin-2 RNAi for fst, inhibitin, wnt-1, and wntless, while nlg-1 looks substantially higher.

There is some variation in the expression of the genes between wounds even in control animals. We repeated this experiment and are including several images of these WI genes here to demonstrate variation between our two samples.

Our interpretation is supported by the fact that none of these genes were significantly different in expression by bulk RNA-seq, at the wound induced time points of 6 hpa and 18 hpa (As shown in Table 2). Bulk sequencing averages the variability across animals, and is a linear amplification technique that would be more quantitative than FISH.

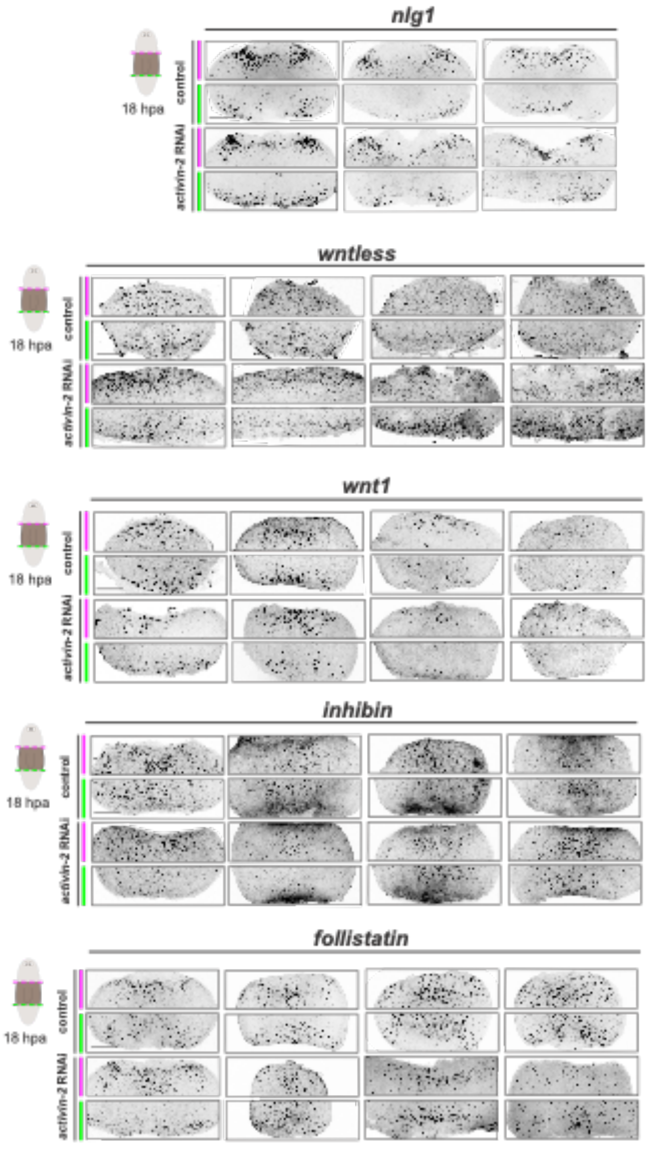

7. The control and activin-2 RNAi images in Figure 5B are placed in opposite order to the rest of

the figures (i.e. control on the right instead of on the left), which is confusing.

Thank you for pointing this out, we modified this figure to have the control animals on the left.

Reviewer #3

In the manuscript entitled 'activin-2 is required for regeneration of polarity on the planarian anterior-posterior axis' Cloutier et al. report a very inspiring phenotype obtained after silencing Activin-2 in planarians. The authors show that activin-2 RNAi resulted in the regeneration of ectopic posterior heads following amputation. Importantly, they observe that notum, the main element of the Anterior signaling center, is not downregulated at 18h in P wounds, providing a molecular explanation for the ectopic posterior-heads. Activin-2 RNAi animals also showed AP axis splitting, and this was specific of regenerating animals, as it did not occurred during normal homeostasis. The authors conclude that Activin-2 could be one of the signals coming form the pre-existing tissue that controls notum expression (Wnt signalling levels) and thus to date it would be the earliest known step in establishing head-versus-tail identity.

The study is of general interest, as it boards general and important questions related with regeneration and tissue patterning. The experiments are properly planed and in general well exposed and justified. However, some interpretations could be misleading, and a deeper analysis and discussion of the main finding, that is, the maintenance of notum expression in P and the regeneration of poles with different identities, should be performed.

- The authors show that in Act-2 RNAi animals notum is not downregulated at 18h in P, and propose that it could be the cause of the multiple heads/tails in P. They also have some evidences that it could be related with the integrity of circular and longitudinal fibers, according to previous published results (Scimone et al. 2017). However, the present study lacks a more in deep analysis and discussion of the mechanism underlying this phenotype. How can it be that from a homogeneous expression of notum in the 18h P wound, few hours later different A and P organizing centers appear?

We appreciate the suggestion and have now endeavored to add new analysis and discussion on this topic. We propose that ectopic wound-induced *notum* expression can result in some anterior PCG activation, and that this can in some but not all cases lead to tipping points of local stable anterior identity. Why stable anterior PCG activation appears localized to only a region of the posterior-facing blastema and in only some animals is not fully understood. However, one possibility is that there is variability in how much anterior PCG activation is caused by ectopic *notum* expression at posterior-facing wounds. Local tipping points could then be stochastically reached with sufficient anterior PCG expression resulting in more anterior PCG expression in new muscle cells. Such a runaway, self-reinforcing process could ultimately be stabilized by formation of an anterior pole from neoblasts choosing an anterior pole fate near anterior PCG expression foci. With a nucleated anterior pole, local posterior PCG expression inhibition and stable anterior PCG expression would occur. In many cases, a tipping point would not be reached, with posterior PCG expression dominating the entire wound and no ectopic anterior pole(s) forming. *notum* does not turn on as early at *activin-2* RNAi posterior-facing wounds as it does at wild-type anterior-facing wounds, possibly explaining why this process is less robust than wild-type head formation. There could also be additional mechanisms that distinguish anterior- and posterior-facing wounds. Regardless, posterior pole formation occurs concurrently with ectopic anterior pole formation in essentially all cases – anterior identity foci formation might only be compatible with posterior pole formation when it is spatially separated from it.

We added this discussion to the manuscript discussion section (Lines 472-494). Note that additional results relevant to understanding the order of events at these wounds are described in the manuscript and in answer to the next review question below.

First, it needs a more detailed description and quantification of the phenotypes, and second, some more experiments could be performed in order to explain how the increase of notum in P at 18h leads to multiple organizing centers with different identity. For instances, the timing of expression of not only notum but wnt1 during P regeneration (only 48 h are shown) could give some clues, as well as the analysis of the longitudinal and circular fibers at the region that must regenerate.

We performed a variety of new experiments and analyses, and the findings are described in this section of the results:

" At 30 hpa, *notum*+ and *wnt1*+ cells were present in variable, but intermingled distributions at posterior-facing wounds (Figure 6C, S7A). At this timepoint positive cells could include both pole progenitors and cells with residual wound-induced expression. Foci of cells reflecting new poles were not yet present. Despite the largely dispersed and intermingled pattern of cells expressing these genes at this early timepoint, two distinct foci of either anterior or posterior pole cells formed later in regeneration.

By 36-38 hpa the anterior PCGs *ndl-5* and *sFRP-1* were ectopically expressed in a locally clustered manner at posterior-facing wounds, prior to substantial ectopic anterior-pole coalescence (Figure 6D, S7B). *notum*+ cells were present at this time, and localized to the regions of *ndl-5* and *sFRP-1* expression. However, there were few *notum*+ cells and they were not yet coalesced into tight foci reflecting new poles. At this time (38 hpa), posterior PCG expression (*wntP-2*) was still broad at the wound, but reduced in level locally in the region of ectopic anterior PCG expression clusters (Figure 6D, S7B). The *notum*+ cells at 36 hpa were *foxD*+, indicating that they were pole progenitors and/or pole cells (Figure 6E). *wnt1*+ cells at this time were also regional and no longer intermingled with *notum*+ cells. Instead, local and separate locations of *notum*+ and *wnt1*+ cells at the same wound face were emerging (Figure 6E). In summary, by around 36 hpa ectopic local anterior PCG expression in *activin-2* RNAi animals was associated with local reduction in *wntP-2* expression and early stages of ectopic anterior pole formation spatially separated from posterior pole cells.

By 48 hpa both *notum+; foxD*+ anterior poles and *wnt1*+ posterior poles showed increased emergence at different locations (Figure S7C). By 72 hpa, all notum+ ectopic anterior-pole cells at posterior-facing wounds had coalesced and the ectopic *ndl-5*+ regions were expanded and stronger (Figure 6D). At 5 dpa, regenerating animals still possessed a global posterior *wntP-2*+ zone, with *wntP-2* expression being only locally cleared near local anteriorized regions (Figure 6F). Reduction of *wntP-2* expression near anterior PCG foci was stronger at 5 dpa than when initial anterior PCG expression was detected at 36 hpa."

We also performed a muscle antibody staining (6G10) on 48 hpa wounds on 21 day and 35 day animals to assess for wound closure, and observed intact muscle at wounds and did not observe any additional aberrations of note beyond descriptions previously in the paper. This finding was incorporated into the manuscript as a part of supplemental Figure S6E.

-In the abstract it is stated that 'Activin-2 is required for this head-versus-tail regeneration decision'. And this appears to be a main conclusion of the study. However, the phenotype shows that Activin-2 seems to be required to restrict a unique axis, but not to decide the identity of the poles. The results show that notum is not downregulated in P, but in fact a tail is regenerated. The interpretation of the results should be more linked to the real observations.

We modified this wording to read: "We report that inhibition of *activin-2*, which encodes an Activin-like signaling ligand, resulted in the regeneration of ectopic posterior-facing heads following amputation."

This was associated not just with ectopic wound-induced *notum* expression at posterior-facing wounds, but also the formation of anterior poles and heads at posterior-facing wounds, concurrently with posterior pole formation and tail formation at posterior-facing wounds.

-In the first section and the corresponding Figure 1 the authors describe the appearance of a 'variable numbers of heads and tails in fragments with both head and tail amputated'. And they show a quantification in Figure 1C. The description and the quantification of the phenotypes observed must be more specific to really understand what is happening in Act-2 RNAi animals. What are really the buds they have in P, or in lateral positions? (SF1). They need to use markers of P and A identity. And how many tails, heads, o tail and heads, appear in P? 2, 3? When there are 3, the one in the middle is always P and the 2 lateral are A? When there are 2, each one has different identity? It's necessary to show a detailed description and quantification to clarify this point, since it's important to understand to which extent Act-2 has a role in polarity, or has a role in controlling notum, or in restricting the P organizing center… In fact, these are possibilities that are not properly discussed in the manuscript.

The animals quantified in 1D represent 2 cohorts, 1) regenerated animals after 14 dpa and 2) uninjured animals, which has now been further specified in the figure.

The 14 day regenerated cohort of animals were quantified using live imaging. Thus anatomic markers are the only data we have on this cohort (which was later put into different batches of FISH), using ectopic eyes as a proxy for head formation and without a clear metric to tell tails from bulges of tissue – we did not quantify # of tails in each animal at this stage. This is now better described in the figure legend.

By live imaging, at 21 days 2/15 had 2 ectopic posterior heads and 13/15 had one – while one animal (shown right of 1B) had an ectopic head surrounded by 2 bulges. At 40 days 4/14 animals had 2 ectopic posterior heads and 10/14 had a single one. Animals at this timepoint were also much more irregularly shaped with significant bulging as seen in Figure S2B. The above quantifications have been added to Figure 1D.

As this bulging correlates with animals that are 54 days from beginning *activin-2* RNAi treatment, one could assume the increased bulging could the result of homeostatic turnover as described in 60 day animals in S3G-I.

These questions are also addressed by data in Figure 6B, we describe that all regenerates stained for *wnt1* and *notum*, possessed one and only one posterior pole. Furthermore, not all irregularities in shape post regeneration appear to be associated with an ectopic pole as seen below; *notum* (green) and *wnt1* (magenta) in a 21d *activin-2* RNAi animal +14 dpa where the posterior pole is offset to one side. Because of this we have tried to limit our interpretation of tail to where we have clear *in situ* data and not only morphology. In sum, the only configurations we have noted using FISH markers are tail alone; 1 tail and 1 head, and head(L)-tail (mid)-head (R). We added this observation to the text.

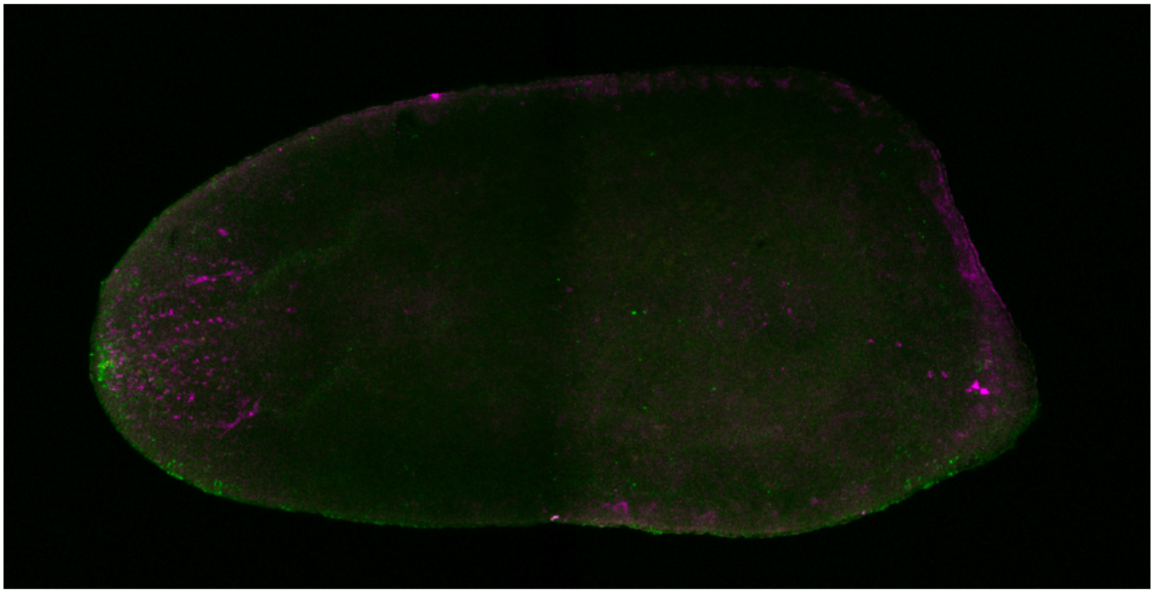

-In the quantification in Figure 1C it seems that A axis bifurcation takes place much later than P axis bifurcation. Why is it like that? In fact, in Figure 6A it is shown that at 72h notum expression is already splited in 2. Thus, why in the graph in Fig 1C there are so few bifurcated heads at 14-21 days? And why the number of splitted A heads increase with time? It should be discussed.

We think our labeling of Figure 1C may have caused some confusion. The first two rows were assessed at 14 dpa, whereas the last row was assessed in intact animals. 21 days and 40 days refers to the number of days on *activin-2* RNAi prior to amputation. We updated the labeling of Figure 1C to reflect this. The intention was to show that animals have different regenerative outcomes depending on the amount of time they have been fed *activin-2* dsRNA, not on the amount of time post-amputation. This comment helped us clarify the labeling in that figure panel.

-Supp Fig 1B- In this experiment the animals have been regenerating for 14dpa, but they have been inhibited for 40 days, so the effect seen in the pharynges are due to tissue renewal, not to tissue regeneration. May be also the lateral buds. When analyzing regeneration, the timing of RNAi and amputation must be taken into account, otherwise one could take wrong conclusions. Furthermore, what is the identity of the lateral buds? Is this a common feature of the phenotype?

We believe ectopic pharynges can be observed in uninjured animals undergoing tissue renewal and in animals undergoing regeneration as we have now added to Figure S2D. We concur that this indicates this can be a consequence of events happening in tissue turnover.

In order to investigate the identity of lateral bulging/budding in intact animals, we analyzed animals at 60 days of *activin-2* RNAi, including with live images. These animals become difficult to incorporate into certain analysis as they have significant change in body shape with a high degree of variation, including irregular tissue growth that no longer preserves their 'flatness' making it difficult to quantify PCGs, etc in FISH.

We do however have PCG stainings indicating that *notum*, *ndl-3,* and *wntP-2* expression are maintained in the correct order in these animals, and without obvious ectopic foci in lateral buds formed at this time, although gradient length quantification is difficult because of the variability in animal dimensions. *chat* was also present in the expected AP distribution without obvious ectopic brains. We therefore suggest that these lateral buds may be the result of local disorganization of muscle and tissue proportions, rather than the result of formation of an ectopic axis.

We incorporated this data into new panels Figure S3G-I.

-The finding that during homeostasis polarity is not affected is very relevant, and it is not properly discussed.

We concur on this point, and we added new data demonstrating this is also true at 60 days of RNAi (S3G-I) and added more discussion of this topic. For context, we compare the results on polarity and PCG expression status in intact animals to what occurs following *β-catenin-1* RNAi. We then suggest that *activin-2* may use a different mechanism than homeostatic Wnt inhibition to achieve changes in polarity, and this mechanism seems to be specific to regeneration (Lines: 215-224). We also discuss these findings in the discussion, and have revised our wording there as well.

-The analysis of the RNAseq is confusing. 14 genes displayed significantly different expression at 6 -18 hours but the authors argue that the analysis by FISH shows no differences. Where is it this FISH analysis? The genes in Figure 3D do not correspond to the ones in Table 2. And furthermore, if RNAseq analysis shows a differential expression, this result is more quantitative than a FISH, isn't' it?

We agree that RNAseq can often be more quantitative for some analyses, but sometimes can be challenging if expression levels are low or noisy, if a phenotype is partially penetrant (with some animals strongly affected and others not), or if a gene is expressed in multiple locations – where FISH gives more spatial specificity and individual animal resolution, making these approaches complementary. Initially we utilized permissive thresholds in hopes of finding other downstream players that may be controlled by *activin-2* that affect polarity through follow-up studies (FISH, RNAi). However, for analyzing the data for wound-induced gene differences specifically, we now re-analyzed the RNA-seq data using a previously utilized threshold for significance of differences in wound-induced gene expression of padj<0.001. This analysis yields six WI genes that meet significance, with *notum* demonstrating the greatest fold change and smallest padj of the 6. We now include FISH for two other genes on this list, which interestingly showed a difference at 18 hpa (of the other three, one did not yield clear signal and for two we could not clone). We included these results as figure S4C, and these genes could be interesting targets for future work.

We modified the wording in the text to reflect that although *notum* is the top gene changed, other tested WI genes that made this list were different by FISH (Lines 263-275).

-In Figure 3D, the authors conclude that there is no difference in the expression of those genes, but apparently wnt1 and wntless seem to be downreglated in P wounds in Act-2 RNAi animals. This result would be important for the study, since upregulation of notum could came together with downregulation of wnt1. This is a very important point that should be clearly solved.

We feel this is largely explained by natural variation in the expression of wound-induced genes from animal to animal, which is seen even in control animals. For thoroughness, we repeated this experiment an additional time, and included several images of these WI genes here (below) to demonstrate variation between our two samples.

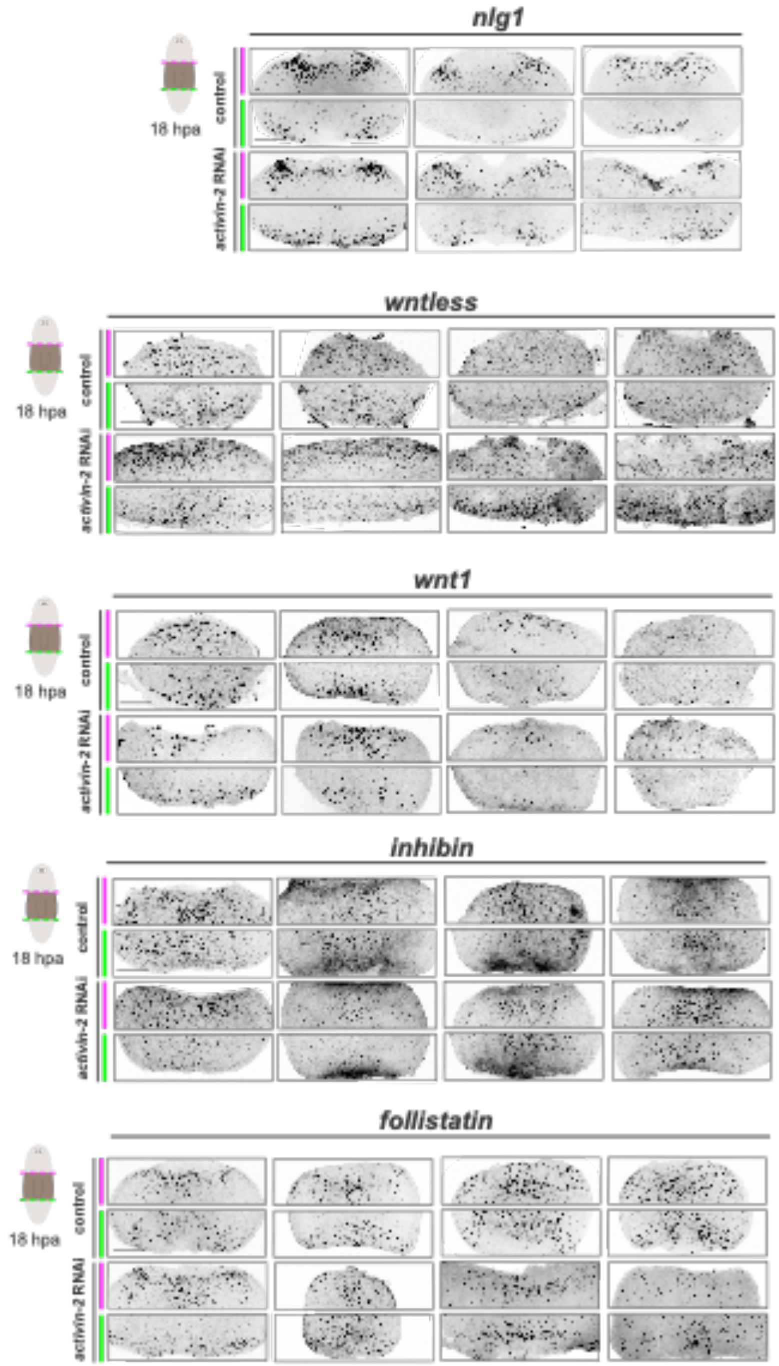

These findings are corroborated by the RNA-seq analyses that show that these genes were not significantly different at the wound induced time points of 6 hpa and 18 hpa (as shown in Table 2).

-The conclusion of the RNAseq section is that ‘Of all wound-induced genes assessed by FISH and RNA sequencing, only notum was affected at the time point when AP regeneration polarity defects emerged following activin-2 RNAi’. This is a strong conclusion that, according to the previously exposed, lacks more supportive data.

We modified the wording in the text to reflect that although *notum* is the top gene changed, some other WI genes that made the significance threshold cutoff were different by FISH.

-The RNAseq results show a very interesting result: notum appears to be expressed in P, and thus, coexpressed with wnt1, but it needs to be downregulated at 18h to make a tail. This result must be discussed.

In Figure 4 we show a timecourse of *notum* expression dynamics in control and *activin-2* RNAi animals (previously in the supplement). The timecourse in the control is consistent with prior studies on *notum* as well (e.g., Petersen 2011; Wurtzel 2015). In the control, *notum* is expressed at much lower levels at posterior-facing wounds than at anterior-facing wounds from the initial phases of expression (by 6h). That said, there is some expression of *notum* at posterior-facing wounds (and homeostatically in the posterior of animals). We didn't compare anterior and posterior-facing wounds in the wild type by RNAseq at this early timepoint here, but that has been done before. The *activin-2* RNAi phenotype (FISH/RNAseq) does indicate that higher levels of *notum* than normal at posterior-facing wounds at 18h can result in an ectopic head nucleated in addition to a tail. We discussed these details more extensively now in a modified discussion.

- The authors show that the loss of notum polarity in activin-2 RNAi animals was observed at 21 but not at 7 and 14 days post-RNAi initiation. They hypothesize that ‘activin-2 could be required during muscle cell turnover to maintain regeneration polarity.’ What does it exactly mean? That Act-2 could be necessary for maintenance of the longitudinal/circular fibers integrity? In fact, the defects observed during homeostasis could fit with this hypothesis. But then, to test if this is true the authors should 1) see if after 7 -14-21 days of RNAi, the mRNA levels of act-2 are really downregulated at the same levels, and if it’s the case, then 2) analyze whether the longitudinal/circular muscles are differentially affected in the 3 situations in the region that will be amputated.

We propose that new fibers display the defect in polarity, and not fibers existing prior to RNAi. The irradiation experiment, which has the same RNAi regime, argues against the *notum* defect seen in this RNAi regime just needing time to work. This experiment suggests it also needs turnover.

As another way to test this possibility, we performed a *myoD/activin-2* double RNAi, and saw that *myoD* RNAi suppressed the *activin-2* RNAi phenotype. *myoD* RNAi blocks production of new longitudinal muscle cells, where *notum* is expressed. As expected, *myoD* RNAi decreased overall wound-induced *notum* expression as was shown in Scimone et al 2017. Interestingly however, *myoD* RNAi depleted posterior *notum* in *activin-2* RNAi animals at a much greater proportion, which is consistent with the cells ectopically expressing *notum* at posterior-facing wounds being newly formed longitudinal fibers.

qPCR for *activin-2* was performed in order to demonstrate that this effect was not simply caused by dilution of the dsRNA. We incorporated these findings as Figure S5F and Figure 5D.

We also performed qPCR on cDNA collected from animals at 7, 14, and 21 days of RNAi as suggested, and saw that *activin-2* was significantly decreased at all time points.

The irradiation experiments do not seem to clarify much the mechanism. In Figure 5B- is really the first image Act-2 RNAi, or it is the control, as in the rest of images? In any case, the conclusion is that notum is still expressed after 6000 rads in P, so at 16h the expression of notum does not depend on neoblast. This could be expected. At this timepoint some notum expression could be neoblast dependent and some could be neoblast independent. But what happens at 18h and later? This should be analyzed. Then after several days of low irradiation notum in P is not expressed anymore in act-2 RNAi animals, but what is the conclusion? That a healthy muscle is necessary to regenerate? How is the muscle in these animals? How are the other organs? May be the digestive system is the one related with notum expression, since irradiation affects all tissues.

Note there were a couple of typos here: We modified figure 5B so that the control is on the left, and 16h has been changed to 18h. We did these experiments at both time points, but included the 18 hour time point because the rest of our data was performed at this timepoint.

We hypothesize that it is newly formed muscle under *activin-2* RNAi, rather than pre-existing muscle, that shows ectopic *notum* expression. This would indicate *activin-2* impacts new muscle differentiation to be polarity competent. Long-term low-level irradiation would reduce new muscle formation over time, explaining its effect. Acute irradiation would have little to no effect because nor muscle turnover happened in this short time window. It is true that irradiation is an imperfect tool, and could have unforeseen side effects on animal biology. However, our hypothesis is also supported by the observation of *notum*+ EdU+ (new muscle cells) cells at posterior-facing wounds of *activin-2* RNAi animals. Furthermore, to address this comment we performed an alternative experiment to demonstrate newly formed longitudinal fibers are required for posterior facing *notum* in *activin-2* RNAi animals.

We performed a *myoD/activin-2* double RNAi, and saw that *myoD* RNAi suppressed the *activin-2* RNAi phenotype. This is described in the prior point above as well. This effect is predicted by the hypothesis because *myoD* RNAi blocks new longitudinal muscle fiber formation, where *notum* is expressed. *myoD* RNAi depleted posterior *notum* in *activin-2* RNAi animals at a much greater proportion which is consistent with these cells being newly formed longitudinal fibers. qPCR for *activin-2* was performed in order to demonstrate that this effect was not simply caused by dilution of the dsRNA. We incorporated these findings as Figure S5F and Figure 5D.

Additional comments:

Lines 125-126. The authors refer to Kenny et al. for the classification of Activin-2. However, in this study Smed Activins are not included. A specific phylogegentic study of Smed Activins/myostatins should be cited or performed. It is important to be clear about the identity of the activin-2 that is the focus of the study. Even more, considering that in the introduction a comparison with activin/follistatin function in other systems is exposed, to suggest its function in animal regeneration.

We performed Bayesian phylogenetic analyses, which indicates that Smed-Activin-2 is in a clade with Activin proteins in other organisms. However, this clade also contains a

Myostatin-like protein and therefore do not want to exclude the possibility that *Smed-activin-2* could be derived from an ancestral, related, *Myostatin-like* gene (which also would interact with Follistatin and signal through Smad2/3). In Mus musculus genes that contribute to Activin proteins are called *inhibin* when not dimerized, we have used this nomenclature in the tree. We added the phylogenetic analysis as Supplemental Figure 1, and discuss it in the text.

Lines 134-137- The authors assume that multiple tails or heads appear at P, but in fact at this point of the study any P marker is analyzed, so the identity of the regenerated fragment cannot be really assessed.

We have edited the text to focus on ectopic heads in Figure 1, and add the assessment of preserved posterior identity later on.

Line 166- Figure S3E should be corrected to Figure S1E

Thank you for noticing this typo. The text has been corrected.

Line 192- It reads after 60 days of RNAi but in the figure legend it reads at 40 days. What is the correct?

The line should read 40 days, and has been corrected. We have also added additional data on 60 days to the supplement.

The interpretation of the graph in Figure 3C is really hard.

We have updated this graph to be more easily accessible by simplifying the cartoons that demonstrate the region of tissue collected.

A scheme showing the RNAi and amputation timing of each experiment would be helpful.

As a primer to the study, we have added a cartoon to Figure 1A describing the general RNAi and amputation timing schematic used in this study.

Do changes in cell death or proliferation could give some clues about the function of Activin-2 in axial restriction?

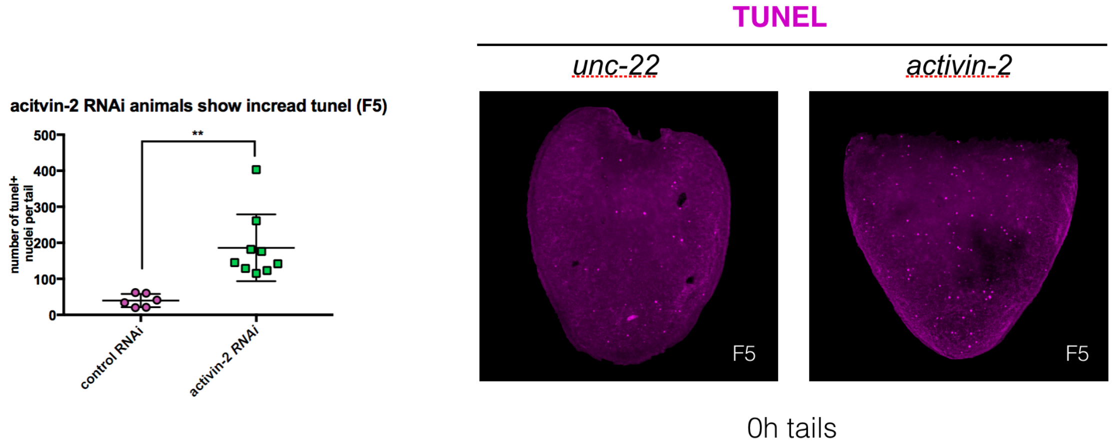

We quantified TUNEL in uninjured (t=0) animal tails and do see a significant increase in *activin-2* RNAi animals. We decided to not include this in the manuscript however, because at this point the underlying basis for this increase is not understood. This is an interesting topic that could be further explored in the future.

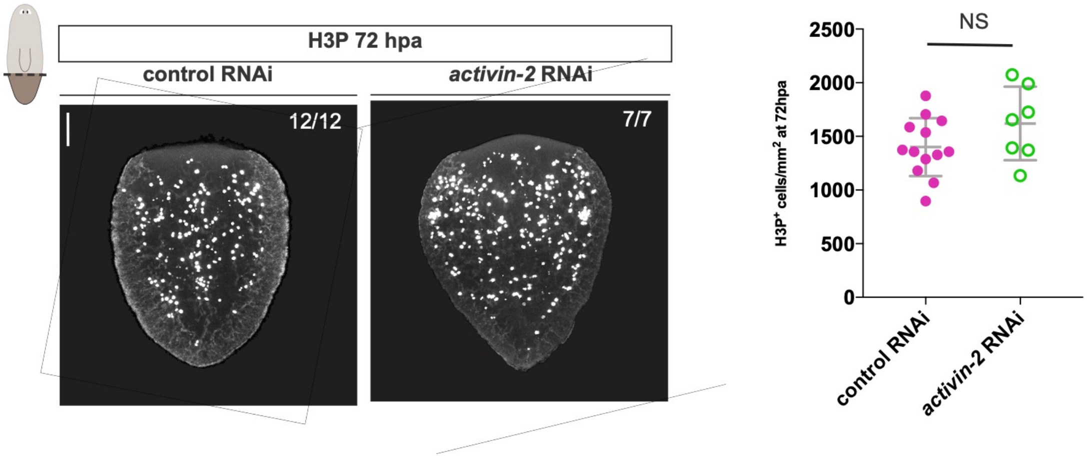

We also quantified H3P at 72 hpa in tails, and with the power of this experiment do not currently see a significant difference between control and *activin-2* RNAi. We decided not to include this result in the paper; the data could be trending that *activin-2* has increased H3P and it would require further experimentation to fully assess this possibility and its possible significance.
