# Supplementary material for: activin-2 is required for regeneration of polarity on the planarian anterior-posterior axis
Source: PLoS Genet. 2021 Mar 29;17(3):e1009466. doi: 10.1371/journal.pgen.1009466 (PMC8057570; doi:10.1371/journal.pgen.1009466)
Supplement: Attachment — Submitted filename: Cloutier_Reviewer reply.docx [file pgen.1009466.s013.docx]

**Reviewer #1:**

Most concerns from the prior review were adequately addressed.

Minor concerns:
1. I think that the authors have made some effort to put the work into prior context. I do recommend that the authors clearly state that their activin-2 is the same as activin in a prior publication, so that readers could better evaluate how this paper fits with prior results and models. It should not be incumbent on the reader to look up sequences and supplementary information to sort this out.

**We have added line 139-140 to the results stating: *activin-2* RNAi of *activin-2* (known as *activin* in (27)) was previously shown to suppress the *follistatin* failed regeneration RNAi phenotype (27, 28).**

2. The authors did add a concentration of dsRNA but not a total volume. Can the authors please add whether 5-8 ug/ul is the concentration of the final liver+dsRNA mix or the initial concentration of dsRNA. If the latter, then the reader may also need to know what volume of dsRNA and what volume of liver mix were used. With the methods as written, an interested reader would not be able to replicate this experiment.

**The prior version stated that the liver to dsRNA mixture was 1:2 by volume, we have added microlitre amounts to the description to further describe the amount of dsRNA used per experiment.**

**Reviewer #3:** In the revised version of the manuscript Cloutier et al. have properly answered and addressed the main concerns. The manuscript has improved substantially by including more detailed analysis of the phenotypes, which were necessary for their understanding. The study is now complete, although the interpretation of the data remains confusing:
1- The result sections include several paragraphs that could be integrated into the discussion. This would make it easy for the reader to follow. Also to simplify it, the last section of the results (Local PCG expression changes in activin-2 RNAi animals were associated with ectopic anterior neoblast fate specification) could be moved to supplementary and integrated with the previous one.

***We changed moved several points discussing implications of data from the results to the discussion as suggested.**

**We integrated the last results section into the prior results section as suggested. With the figure, instead of moving it to the supplement, we improved figure layout so that it fits better graphically. We found these results significant enough to still merit inclusion in the main figure.**

2-The results indicate that Activin-2 is restricting notum to the anterior wound at 18h, which is a key stage to specify the identity of the pole in the wounds. The co-expression of wnt1 and notum in posterior wounds leads to the range of defects in posterior regeneration described in the study. The problem is the conclusion about the mechanism of action of Activin-2 . The authors conclude that Activin-2 could be necessary for the establishment of a ‘polarity’ in the longitudinal fibers, which are the ones expressing notum. The main reasons underlying this conclusion are 1) that according to their interpretation, the muscular fibers do not appear affected in the activin-2 RNAi animals; and 2) inhibition of myoD+Activin2 avoids the ectopic expression of notum in posterior. However, 1) in Supp Fig 6 E the muscular fibers do not seem properly organized at 48h of regeneration in Activin-2 RNAi animals (in 21 days RNAi animals they could already be affected according to supp figure 3D). Regarding point 2) myoD inhibition decreases notum expression in anterior and posterior wounds. The quantitative difference between them could be a matter of timing, since, as the authors explain, the expression of notum in posterior wounds is delayed with respect to anterior, and this is a crucial point in the current interpretation of the data. According to the data, an alternative explanation could be that activin-2 is required for the proper regeneration of the muscular fibers (longitudinal and/or circular) and not for the establishment of a supposed polarity. This would also explain the duplication of poles seen in anterior wounds.

**On point 1, we think the most relevant analysis of fiber orientation is prior to amputation, rather than during early stages of blastema formation (the 48h fig S6E image) because *notum* expression at wounds occurs in the 6-18h wound-induced phase. RNAi of *activin-2* led to fiber disorganization at late stages (40d) of homeostasis in the RNAi timecourse. When amputations were performed at early *activin-2* RNAi timepoints (21d) fibers were not visibly aberrant in intact animals and the *notum* asymmetry phenotype was present. At this point we don't feel comfortable making strong conclusions about morphology of muscle at the 48hpa timepoint in comparison to control being normal or abnormal. We simplified this to just now make the simplest statement that we feel can be said about this 48hpa timepoint that muscle was present. This timepoint is after the wound-response phase so we feel that characterizing the dynamics of muscle repair/growth in blastemas is not really the place to focus.**

**Importantly, we do describe a muscle organization defect, but this was observed after the *notum* phenotype. It could be that if muscle polarity is being perturbed, ultimately this could impact muscle pattern. In our discussion we do not mean to propose a nature for the defect in longitudinal muscle that affects *notum* asymmetry that would exclude a number of possibilities such as this or others. We added text to the discussion to try to be clear on this point:**

"Inhibition of Activin could perturb the polarization of newly synthesized longitudinal muscle or a different process essential for the capacity of longitudinal muscle to properly regulate *notum* activation asymmetrically."

**One other note here is that the expression of muscle genes was not detectable impacted after *activin-2* RNAi - unlike the case following RNAi of muscle development genes *myoD* and *nkx1*. We added this information to the supplement as Figure S4A. We have clarified this point in the text by adding the statement to the results:**

“No significant defect in muscle-specific gene expression was observed at the time of amputation (Figure S4A). This data together with the data that muscle fibers and cell numbers were normal at this RNA timepoint (Figure S3E-F), suggest that the *notum* expression phenotype was not overtly caused by changes to fiber number or morphology."

**Finally, note that *activin-2* RNAi does not impact the levels of *notum* expression (or other muscle wound-induced genes) at anterior-facing wounds indicating that longitudinal fibers are present and capable of normal levels of wound induction as shown in Figure 3B.**

**On point 2, regarding timing in the *myoD* RNAi experiment we looked at *notum* expression asymmetry at 18hpa; after this time, wound-induced expression in general wanes. Therefore, we feel we looked at the suitable timepoint to assess whether *notum* expression asymmetry would be present. Note that *myoD* RNAi blocks new fiber production and *myoD* RNAi itself does not detectably affect *notum* expression asymmetry (anterior-vs-posterior wounds) in remaining fibers.**

**We added text to explain this better:**

"Perturbing muscle production, such as with *myoD* RNAi, does not cause loss of *notum* expression polarity in remaining fibers; furthermore, longitudinal fiber number was normal in *activin-2* RNAi animals with polarity defects."

**Therefore, the *myoD* RNAi experiments support a conclusion that the *activin-2* RNAi phenotype results in *notum* expression at posterior-facing wounds in new fibers (this is also supported by the irradiation experiment and EdU experiments in the paper).**

**Overall, we propose that *activin-2* is required for new longitudinal fibers to be competent to display regeneration polarity (asymmetry in wound-induced *notum*). What aspect of muscle development is affected is unknown - it could be a polarized process in longitudinal fibers or some other process that impacts the ability of these fibers to display a polarized response is unknown. However, general fiber production (number of cells, maturation of muscle cell phenotype) was not aberrant and generally affecting muscle production (*myoD* RNAi) does not cause loss of *notum* expression polarity**
